# Increased Accessibility to Primary Healthcare Due to Nurse Prescribing of Medicines

**DOI:** 10.3390/ijerph19010292

**Published:** 2021-12-28

**Authors:** Dorota Kilańska, Anna Lipert, Marika Guzek, Per Engelseth, Michał Marczak, Kamila Sienkiewicz, Remigiusz Kozłowski

**Affiliations:** 1 Department of Coordinated Care, Medical University of Lodz, 90-131 Lodz, Poland; dorota.kilanska@umed.lodz.pl; 2 Department of Sports Medicine, Medical University of Lodz, 92-213 Lodz, Poland; 3 Medical and Diagnostic Center (MCM), 08-110 Siedlce, Poland; marika.guzek@centrum.med.pl; 4 Narvik Campus, Tromsø School of Business and Economics, The Arctic University of Norway, 8505 Narvik, Norway; per.engelseth@uit.no; 5 Department of Management and Logistics in Healthcare, Medical University of Lodz, 90-131 Lodz, Poland; michal.marczak@umed.lodz.pl (M.M.); kamila.sienkiewicz@stud.umed.lodz.pl (K.S.); 6 Center of Security Technologies in Logistics, Faculty of Management, University of Lodz, 90-237 Lodz, Poland; remigiusz.kozlowski@wz.uni.lodz.pl

**Keywords:** nurse prescribing, nonmedical prescribing, access to health services, task shifting

## Abstract

Since January 2016, nurses and midwives in Poland have had the right, with some restrictions, to prescribe medicines. Consequently, Polish patients received the same opportunity as in other countries worldwide: easier access to certain health services, i.e., medical prescribing. The aim of this study was to assess the impact of structural changes which increased the nurses’ competences on the accessibility to prescription visits for patients receiving primary healthcare on the example of Medical and Diagnostic Centre (MDC), and to discuss the general trend of legal changes in nursing profession regulations. We performed a detailed analysis of the data on the MDC patient population in Siedlce who received at least one prescription written by a general practitioner and/or a nurse/midwife in the years 2017–2019.The largest number of prescription visits made by nurses concerned patients aged 50–70 years, as this age range includes the largest number of patients with chronic diseases who need continued pharmacological treatment originally administered by doctors. An increasing tendency for prescription visits made by nurses was recorded, with a simultaneous downward trend in the same type of visits undertaken by doctors at MDC. Nurses’ involvement in prescribing medications as a continued pharmacotherapy during holiday seasons results in patients having continuous access to medication. An upward trend was also observed in the number of medications prescribed by nurses per patient. Structural changes in the legal regulations of the nursing profession improve patients’ access to prescription visits under primary healthcare. Further research is recommended to evaluate the dynamics of these trends and the impact of newly introduced nursing competences on the accessibility of prescription visits for patients.

## 1. Introduction

Since Declaration of Alma-Ata, innovations in healthcare have been introduced as a response to ageing populations, increased incidence of chronic diseases and multiple morbidities, but also to strongly promote a healthy lifestyle and preventive measures [1]. The assumption was to shift healthcare services from hospitals to local communities, which encouraged countries worldwide to design new therapeutic models in primary healthcare (PHC), which was to become the first point of contact in healthcare services [2]. It was noticed that the tasks carried out by doctors did not always require their medical competence and could be delegated to other medical professionals that were already included in health systems, and whose competences were sufficient enough to provide the necessary scope of service. Regarding prescribing medications, the nursing occupation became one such medical profession [3]. In 2012, the World Health Organization (WHO) adopted a standpoint which emphasized the necessity of task-shifting from doctors to nurses [4]. In its global strategy on human resources for health WHO 2030, the WHO underlined the need to maximize the potential of midlevel practitioners to increase human resource efficiency [5]. Additionally, the use of the potential of nurses was enhanced in global and European strategies, prompting an increased share of nursing and midwifery in the improvement of the health status of the population [6]. The strategies assume that healthcare services provided as close to the population as possible will increase health protection accessibility and improve its effectiveness. Why is healthcare effectiveness significant? The concept, as well as the issue of cost-effectiveness and value for money, aims at capturing the extent to which the inputs into healthcare systems, in terms of expenditure, labour and capital, are used to provide valuable goals for healthcare. It constitutes one of the most widely discussed aspects of healthcare systems’ functioning, including the increase in its accessibility [7].

In 2010, the WHO defined universal health coverage (UHC) [8] “ensuring that all people can use the promotive, preventive, curative, rehabilitative and palliative health services they need, of sufficient quality to be effective, while also ensuring that the use of these services does not expose the user to financial hardship” [9]. In 2019, the United Nations General Assembly meeting entitled “Universal Health Coverage: Moving Together to Build a Healthier World”, aimed at accelerating progress toward universal health coverage, including access to quality essential healthcare services and access to safe, effective, quality and affordable essential medicines including vaccines for all [8]. This implies that the use of these services does not expose the users to financial hardship, with a special emphasis on the poor, vulnerable and marginalized segments of the population [8]. This resolution defined goals which should be achieved to “implement the most effective, high-impact, quality-assured, people-centered, gender-and disability-responsive and evidence-based interventions to meet the health needs of all throughout the life course, and in particular those who are vulnerable or in vulnerable situations, ensuring universal access to nationally determined sets of integrated quality health services at all levels of care for prevention, diagnosis, treatment and care in a timely manner”. The aim of this resolution was also to “promote increased access to affordable, safe, effective and quality medicines, including generics, vaccines, diagnostics and health technologies”. According to the WHO, what it means is that all individuals and communities receive the health services they need without suffering financial hardship. The delivery of these services requires adequate and competent health- and care-workers with an optimal skills mixture at the facility, outreach and community level, and who are equitably distributed, adequately supported and enjoy decent work [8]. Poland is the one of the countries with the highest spending on PHC, which accounts for around 12 percent of all health spending [10]. Every third respondent by CBOS in Poland assessed the availability of a primary care physician negatively [11]. Poland ranks last in the European Union. The report “Health at a Glance 2021”, prepared for European countries by the OECD and the European Commission, shows that basic healthcare was in the most difficult situation. The percentage of family doctors—compared to other EU countries—is dramatically low, and amounts to only 9 percent of all specialists [10]. At the peak of the infection season, access to primary healthcare clinics is difficult, especially in large cities. Doctors not only see patients, but also provide the so-called teleport. In addition, the number of COVID-19 infections is growing rapidly, and it is the primary care physician that people suspecting infection with the SARS-CoV-2 virus seek help from [12]. Coronavirus disease (COVID-19) is an infectious disease caused by the SARS-CoV-2 virus [13]. In recent decades, chronic conditions have increased the demand for healthcare services and increased costs. For the Triple Aim model, countries should find solutions which help to decrease the costs, increase quality of care and help people to achieve better access to healthcare [14]. Nurse-prescribing is a way to save costs for the patient (transport, time and money) and has potential to save costs for the health system. It means that physicians have more time to see more acute and complex patients). The nurses’ prescribing was assessed on the same level as physicians and had a high patient-satisfaction rate [15]. The main reason for task-shifting is because nurses have more time to spend on providing information and counselling for patients [16]. Nurses’ prescribing is a way to increase access, especially in rural areas and provide efficient resource management [17,18]. Task-shifting is one of the solutions which can help with better access to healthcare for patients in all countries, as well as in Poland, and has been identified as a strategy to mitigate deficiencies, improve quality and improve efficiency [19]. The effects of prescriptions by nurses are comparable to those of doctors [20].

Nurse prescribing of medicines pertains to the official right given to nurses to prescribe a selected range of medications or a full list of prescription medications [21,22]. The extent of prescribing medications by nurses depends on several factors: firstly, the category of nurses entitled to medication prescribing that ranges from limited, highly specialized groups to all the nurses with a license to practice a profession; secondly, kinds of medications that nurses have the right to prescribe, from all medications to restricted sets; thirdly, general legal liability, from independent prescribing to the model of delegated prescription under doctor’s supervision [22,23,24]. Nurse prescribing of medications has been demonstrated to be comparable with medicine prescription by doctors, which is evaluated by the number of prescribed medications and the kind and doses of selected medications [20,25,26]. Patients who received prescriptions written by nurses were found to be as satisfied or more content with the intervention provided by nurses than with prescriptions issued by doctors [25]. Cochrane systematic review demonstrated that in numerous disorders, including chronic diseases, nurses were as effective as doctors [26].

The position of Polish Nurses Association (PTP), with contribution from the European Federation of Nurses Association (EFN), was presented at the debate on Advance Nursing held in Polish Parliament. As a result of the meeting, a decision on medication prescription by nurses was taken [27]. At that time, many different statements concerning health-related environments were presented in the media [28,29,30,31]. Many of them were against nursing autonomy in this area. However, several years later, Polish government was looking for solutions to increase service availability for patients, and the Department of Nursing and Midwifery Services in the Ministry of Health put this proposal into consideration. Again, intense discussion broke out about benefits and threats of the solution [19,21,32,33,34,35,36]. The global number of nurses prescribing medications is growing steadily. In recent years, the same trend towards increasing the role of nurses in this respect has been observed in Europe, which is indicated, among others, by the implementation of new regulations on the rights to prescribe medications, whose extent varies between the countries. Most of the countries where the applicable law empowers nurses to prescribe medications have already adopted and implemented relevant regulations within the last dozen or so years. Nevertheless, prescription rights for nurses are still limited in most European countries, particularly the countries which have made such legal amendments only recently. Nowadays, a total of 13 countries in Europe have laws on nurse prescribing in place [37].

With strong support of international organizations from Europe, Polish patients received the same opportunity as in other countries around the world: easier access to healthcare services—medical prescribing—and since January 2016, nurses and midwives in Poland have been able to prescribe medicines with some restrictions. Today, the nurse and midwife may issue up to four prescriptions for consecutive periods of use not exceeding a total of 120 days. In the case of an electronic prescription, nurses and midwives may prescribe a maximum amount of a medicinal produce, food for particular types of nutritional uses or medical devices for one patient at a time, necessary for the patient for a180-day period of use calculated based on the dosage method specified in the prescription, and in the case of medical products, they can be prescribed only as part of a continuation of a medical order. The range of drugs for which nurses can issue prescription and continuation prescriptions excludes all substances belonging to the strong (list A), intoxicating and psychotropic substances. Nurses cannot prescribe prescription drugs and drugs imported as part of target import, either. An additional restriction applies to midwives, as this professional group is excluded from prescribing to seniors 75+ (S) (from 2020). In addition, nurses cannot independently prescribe contraceptives, as they may only be prescribed by nurses for continued use.

The aim of this study was to assess the impact of structural alterations which increased nurses’ competences on the accessibility of prescription visits for patients under primary healthcare, illustrated by the example of the Medical and Diagnostic Centre. The study included an important task of evaluation of how certain demographic indices affected the number of prescriptions issued, the number of medications prescribed as well as a general trend in amendments to the regulations of the nursing profession.

## 2. Materials and Methods

The Act of 15 July 2011 on the professions of nurse and midwife extended the catalogue of people entitled to issue reimbursed prescriptions. Pursuant to the Act, as part of the independent provision of preventive, diagnostic, therapeutic and rehabilitation services, a nurse and midwife with a second-cycle diploma in nursing or midwifery, and a nurse and midwife with the title of specialist in nursing have the right to: prescribe drugs containing specific active substances, with the exception of drugs containing very strong substances, narcotic drugs and psychotropic substances, and food products for particular nutritional purposes, including prescribing them; prescribe specific medical devices, including issuing orders or prescriptions for them if they have completed a specialist course in this field.

As part of the implementation of medical orders in the diagnostic process, treatment and rehabilitation, a nurse or midwife with a diploma of at least the first degree in nursing or obstetrics, as well as a nurse or midwife with the title of specialist in nursing have the right to issue prescriptions for drugs, with the exception of drugs containing certain substances, very strong narcotic drugs and psychotropic substances, as well as foods for particular nutritional uses necessary for the continuation of treatment, if they have completed a specialist course in this field.

The Regulation of the Minister of Health of 18 January 2018 (Journal of Laws 2018, item 299) on the list of active substances contained in medicines, foodstuffs for particular nutritional uses and medical devices ordered by nurses and midwives and the list of diagnostic tests for which nurses and midwives have the right to issue referrals, specifies:

- A list of active substances contained in medicines that can be prescribed by nurses and midwives (groups of medicines, active substances, form and route of administration);
- A list of foods for particular nutritional uses that may be ordered by nurses and midwives;
- A list of medical devices for which nurses and midwives are entitled to issue prescriptions and orders;
- A list of diagnostic tests for which nurses and midwives are entitled to refer.

Currently, a nurse can prescribe 274 medications without the supervision of a physician, excluding medications containing very potent substances, narcotic drugs and psychotropic substances. On the other hand, they have the right to continue all medications provided that a medical order is included in the patient’s documentation, with the exception of medications containing very potent substances, intoxicants and psychotropic substances. The range of drugs for which nurses can issue prescription and continuation prescriptions excludes all substances belonging to the strong (list A), intoxicating and psychotropic substances.

In addition, nurses cannot independently prescribe contraceptives and may only prescribe them for continued use.

From 1 August 2020, as part of primary healthcare advice, a nurse may, inter alia, conduct disease prevention and health promotion, select wound-treatment methods as part of medical services provided by a nurse without a doctor’s order, and prescribe medications containing specific active substances, including prescribing them. However, nurses are not able to prescribe medications containing very potent substances, narcotic drugs and psychotropic substances. Instead, they are able to issue prescriptions for drugs prescribed by a doctor as a follow-up, and issue a referral for specific diagnostic tests.

The data were collected from MDC in Siedlce, a healthcare facility where the first ePrescription in Poland was issued. At MDC, an incentive scheme for nurses has been developed, rewarding the effectiveness in taking up preventive and health-promotion tasks in order to improve quality of services while reducing their costs. The effectiveness of care towards the Triple Aim was also achieved by delegating medicine prescription to nurses, and thus improving access to services for patients. For this purpose, nurses employed at MDC were trained in prescribing medications on courses, and the other prescriptions were available only from the IT system. Nonmedical workers were motivated to improve their competences and complete their nursing studies. Thus, the task of shifting and lean management became feasible by shortening the path of access to prescription services in chronic diseases and making the time obtained in this way available to other patients who, due to the burden on doctors, could not receive the service when they needed it.

### 2.1. Characteristics of the Medical Institution, Services and Patients

The Medical and Diagnostic Centre, established in 1998, is owned by Polish citizens, professionally connected with the healthcare sector (68 shareholders: physicians, nurses, physiotherapists, laboratory diagnosticians, lawyers and economists). The company operates a network of 41 clinics in the central-eastern part of Poland, in the Mazovia and Lublin province. The main areas of activity include the following: primary healthcare (with about 100,000 patients under care),outpatient specialist care, rehabilitation, health-promotion, long-term care (including two nursing homes), psychiatric care with addiction treatment, dentistry, 16 pharmacies (pharmaceutical care unit in progress).

The company, which employs over 600 employees, is a national leader in the field of coordinated care, whose experience has been repeatedly presented at various conferences and meetings in the country and abroad. In 2019, MDC was invited by the World Health Organization to a project on expanded roles of nurses in PHC [38]. Disease-management programs of healthcare delivery for patients with noncommunicable diseases (NCDs) were implemented in MDC in 2015. According to the latest data, there are over 20,000 patients with NCDs under care at MDC. The largest group are the patients with heart failures, arterial hypertension, diabetes, chronic obstructive pulmonary disease (COPD), asthma, and chronic back pain. These patients often suffer from multiple morbidities. The problems typical of older patients, such as dementia, are also becoming more and more challenging due to the ageing population, which also affects Poland. Healthcare mainly focuses on the primary healthcare level, where the GP is a decedent of the patient’s treatment plan, a nurse supports the medical aspects of care, and the healthcare coordinator keeps the process organized for patients and medical staff. The model is based on an annual general examination, at least once a year, and developing the individual diagnostic and treatment plan as well as planning the follow-up/control visits with the frequency adjusted to the patient’s needs and clinical condition. Patients are consulted by the specialists (e.g., cardiologists, diabetologists, etc.) after the GP’s referral. Every medical specialist uses the same EMR (Electronical Medical Record)and can see medical notes made by others, results of the diagnostic tests and all prescribed medications for a specific patient. Since the medication prescription rights for nurses were implemented and electronic prescriptions became available in Poland, the aim has been the takeover of some medical appointments, mainly check-up visits, by nurses from doctors. Thanks to the possibility of prescribing drugs, nurse visits can now be more complex and include education, control of health conditions and parameters, and finally, medication continuation. The purpose of the stratification model of PHC patients is involved identification and isolation of subpopulations with a similar disease severity, and thus with similar needs for medical care.

This allows the development and implementation of an appropriate diagnostic and therapeutic healthcare plan. Thanks to the stratification model, we can estimate the potential costs in the healthcare system due to health risks and the possibility of destabilization. Due to their data interoperability, the data from MDC IT systems were included.

**Figure 1:**
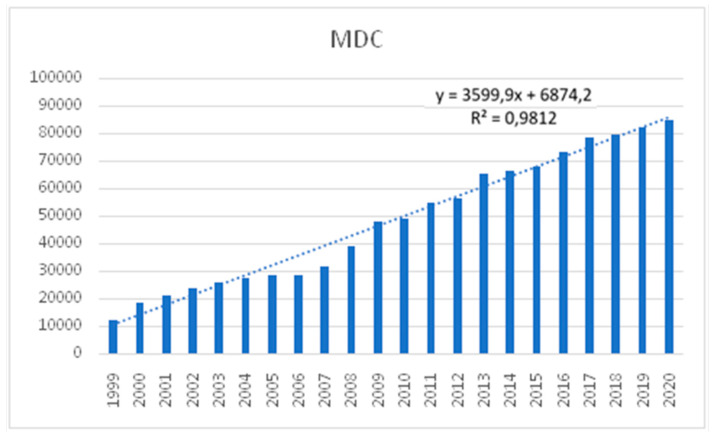
The number of patients receiving healthcare at Medical and Diagnostic Centre (MDC).

The line presented in Figure 1 shows an upward trend. We can therefore predict that the prescription service provided by nurses is likely to gain an increasing popularity and shows an upward, close to linear, trend. The theoretical linear trend model accounts for 98 percent of the variability of the dependent variable in the presented chart.

**Figure 2:**
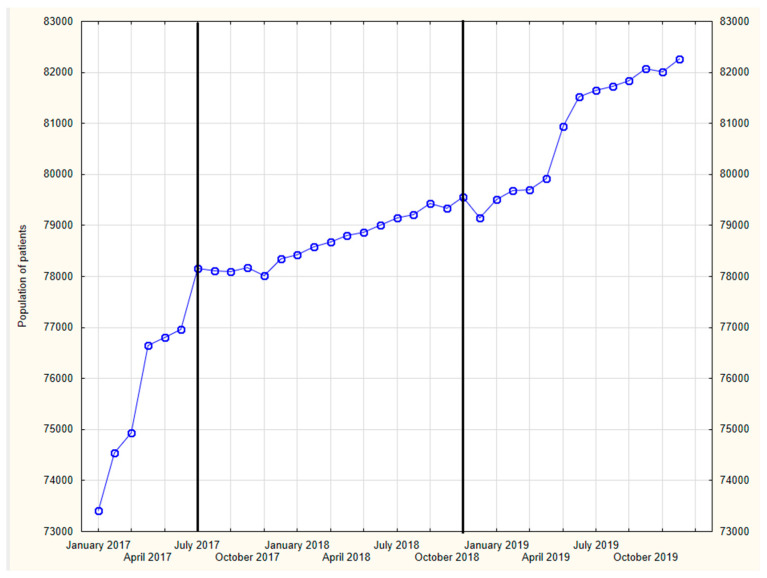
The patient populations from 2017 to 2019.

In our further research, the time span of 2017–2019 shall be analyzed in detail. The number of patients per month at MDC in the analyzed time span is presented in Figure 2.

When analyzing the data shown in Figure 2, three stages of the development of the investigated issue can be distinguished:

1. Rapid increase between January 2017 and June 2017;
2. Stable rise from July 2017 to January 2018;
3. Return to stable increase from January 2018 to the end of the analyzed period in December 2019.

The growing number of patients is mainly related to the establishment of new PHC units, mainly in rural areas in Mazovia province. By 2017, there were 24 PHC units at MDC; as of June 2017, MDC included four new PHC units and by March 2019, another five new PHC units were added.

Figure 2 reflects the dynamics of changes impatient population in 2017–2019. Initially, until June 2017, a more dynamic rise in the number of patients can be observed. This is a consequence of three more primary healthcare centers which were established in 2016. Next, from July 2017 till April 2019, the dynamics of the changes clearly diminished. Afterwards, from May 2019, another stage of sharp increase in the number of patients is seen, and it may be the implication of the establishment of additional two primary healthcare units.

**Figure 3:**
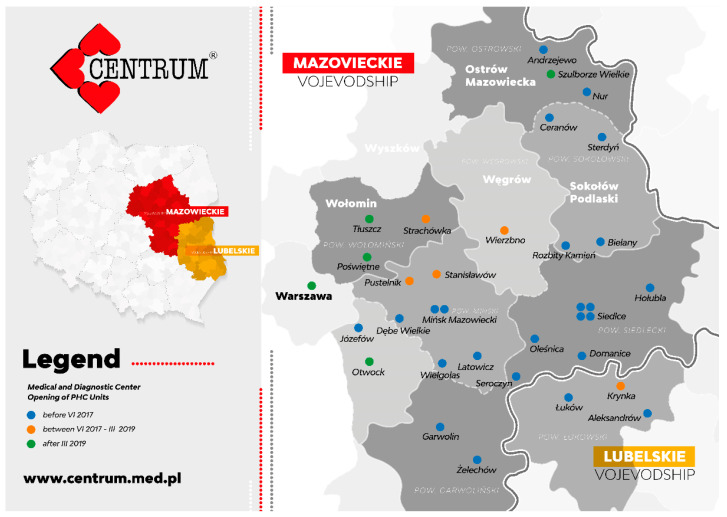
MDC development from June 2017 to June 2019 with the three specified stages.

Newly established MDC units in the specified stages of development are marked in different colors on the map below–Figure 3.

### 2.2. Statistical Analysis

Statistical analyses were performed using STATISTICA (StatSoft, Inc., version 10,2011, Lodz, Poland). The data are presented mainly as number or percentage of cases. To assess the relationship between the number of visits to the nurses or GPs in relation to the number of accessible nurses or GPs, Pearson’s correlation was performed. To statistically separate a limited number of medications which were the most prescribed by the nurses or GPs, Pareto analysis was performed. Time series analysis was performed to present the seasonality of visits to GPs. Significant differences were accepted for all analyses at the level of *p* < 0.05.

## 3. Results

**Figure 4:**
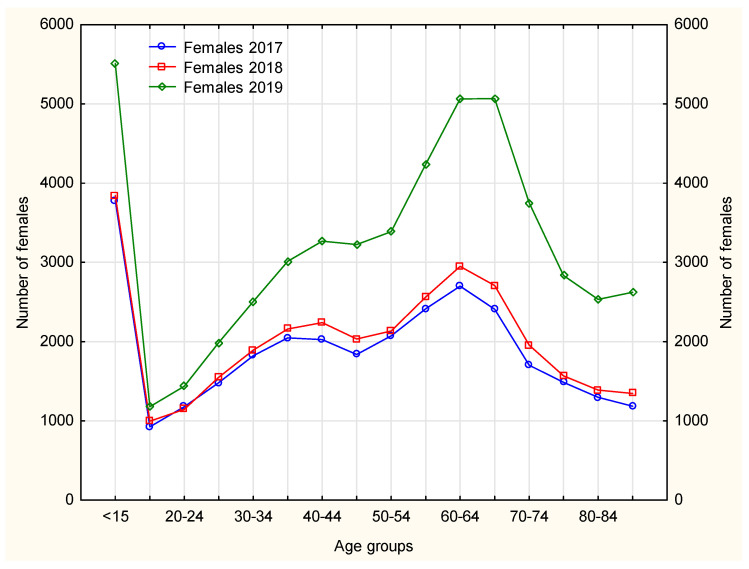
The number of female patients every year separately according to age groups.

**Figure 5:**
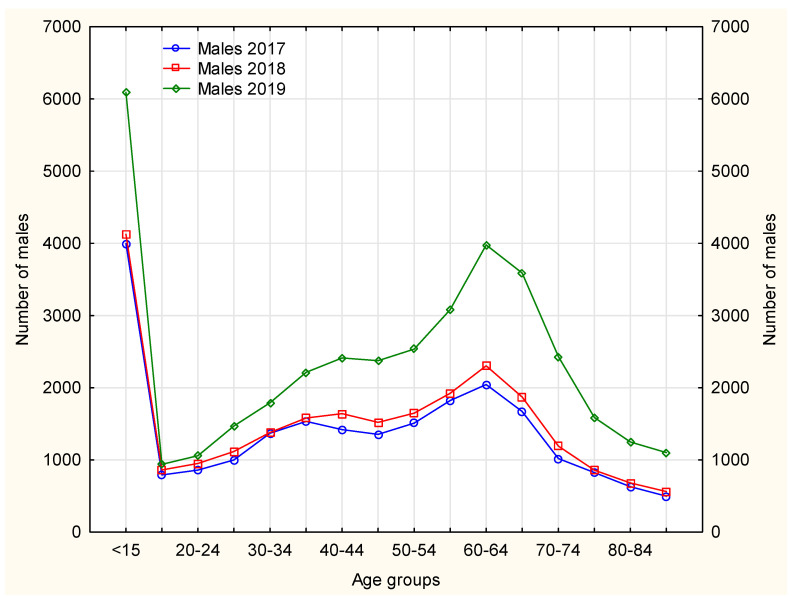
The number of male patients in each analyzed year separately according to age groups.

The nurses mainly handled continuation of pharmacotherapies in patients with chronic diseases, hence the age structure, as the largest number of chronically ill patients fall within the range of older than 50–Figure 4 and Figure 5. Nurse prescribing of medications for this age group, however, causes some difficulty, as some medications cannot be prescribed by nurses (e.g., soporific drugs).

In 2019, among female patients using prescription nursing services, an increase was observed in using this service instead of the doctor’s, thus freeing up space for patients with acute diseases–Figure 4. In the case of Poland this is an important achievement, bearing in mind the number of family doctors available to patients.

**Figure 6:**
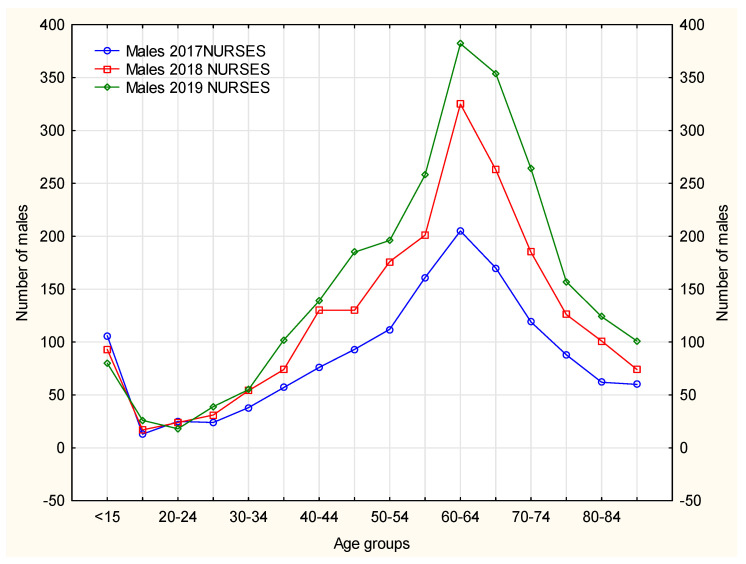
The number of male patients visiting the nurse in each of the analyzed years separately according to age groups.

Females made prescription visits considerably more frequently than males. In 2017–2019, the age groups (both females and males) that received prescription visits most frequently included patients aged ≤15 years and 60–64 years. In 2019, the largest number of prescription visits required by both male and female patients was recorded in patients aged 65–69 years–Figure 5 and Figure 6. The number of prescription visits in male population was lower by one-fifth that of females.

**Figure 7:**
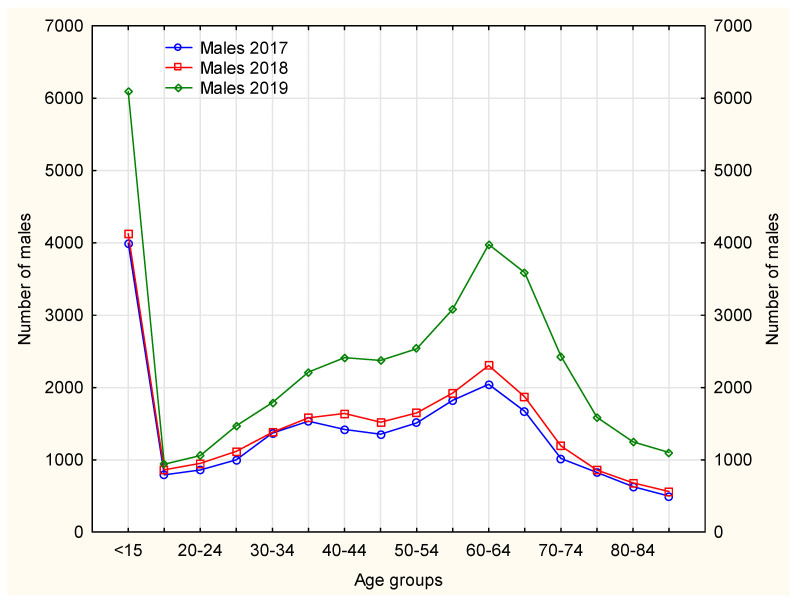
The number of male patients visiting the GP in each of the analyzed years separately according to age groups.

According to Figure 7, prescription visits made by nurses were recorded more frequently for female than male patients. Such medical service was found to be the most popular with females aged 65–69 years. In 2017–2019, in males, prescription visits were required more often by younger patients (60–64 years) than in the group of females. A similar trend can be observed for prescription visits made by doctors. The opportunity of nurses providing prescription visits is gaining an increasing interest. In the analyzed period of time, in the 16–19 years age group the number of prescription visits required by males was recorded at a similar level for both nurses and doctors. It is noteworthy that in 2019, this age group paid more prescription visits to their primary healthcare doctor. This may relate to the scope of practice of the nurses who are not entitled to prescribe medications from the full list of reimbursed medicines. It should be underlined here that the number of prescription visits to the doctor was ten times higher than to the nurse. Males aged 60–64 years, however, used this medical service proportionately more often at the nurse’s office, which was markedly noted in 2018 and 2019.

### Analysis of the Services Used after Changes in Medical Law/Due to the Greater Legal Rights of Nurses

**Figure 8:**
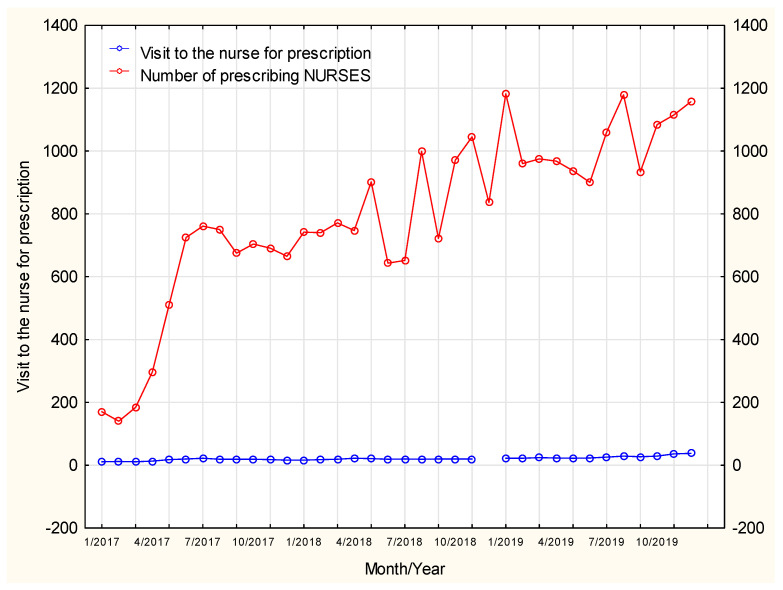
The number of licensed nurses and the number of patients’ visits.

Despite a constant number of nurses with prescription rights, the number of prescription visits shows an upward trend. The number of prescription visits to the doctor was observed to decrease in summer months (July–August) whereas at the same time the number of such visits to the nurse was found to increase. As a result, patients still had the opportunity to continue their pharmacotherapy despite limited or no access to doctors. The number of prescription visits in January, April and November was on the rise both in doctors’ and nurses’ services Figure 8.

The steady increase in the number of prescription visits was not affected by a growing number of patients going on visits, therefore, in this course of time, nurses provided prescription visits increasingly more often. A potential hypothesis may assume that it was related to their greater experience in writing prescriptions and potentially greater trust developed in patients who already had contact with the nurse prescribing medications and used their services as willingly as the services offered by doctors.

A considerable disproportion between the number of prescriptions issued by doctors and by nurses results from the fact that the latter are entitled to prescribe nearly a full range of medications (without list A), exclusively as a continuation of pharmacotherapy in chronic diseases, after a relevant approval by the doctor in the patient’s medical records. The list of medications which nurses have the right to prescribe independently is restricted and specified by law.

**Figure 9:**
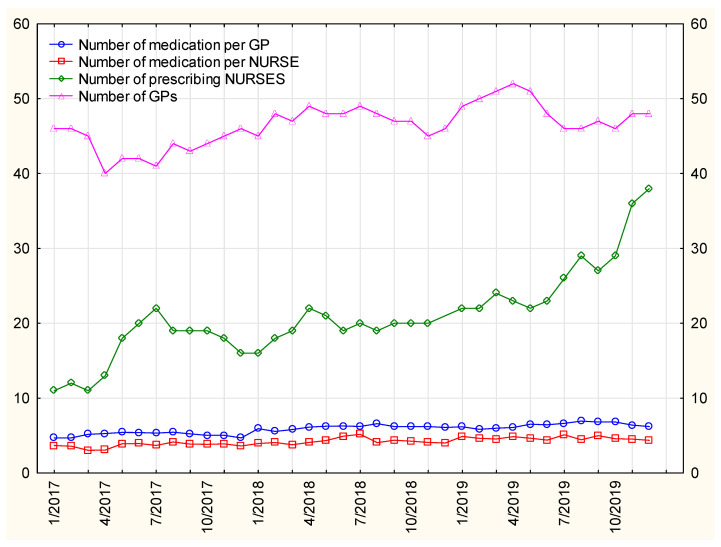
The mean number of medications prescribed by the nurse and GP per patient in relation to the number of prescribing nurses and GPs.

The number of medications prescribed by nurses per person shows an upward trend—Figure 9. This means that prescription visits were not random, one-off visits providing a single-drug prescriptions, but they were complex and complete services when full sets of medications for chronic diseases are prescribed. Nurses seem to have clearly gained more trust.

The number of nurses authorized to continue treatment is systematically increasing, but none of the visits were at the same level, with a specific increase in the level in summer periods, when access to medical services decreased.

**Figure 10:**
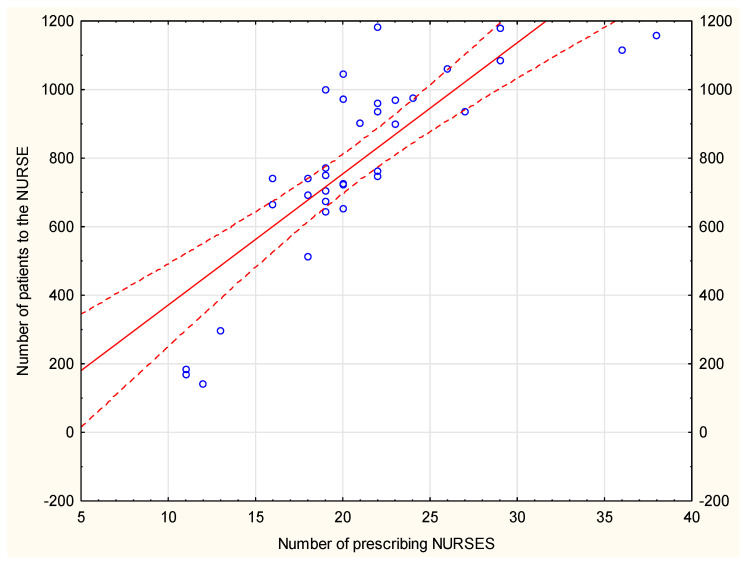
The correlation between the number of patients visiting the nurse and the number of nurses with the license to prescribe medications.

There is a positive and strong linear correlation, with r= 0.81348, between the number of patients and nurses with permission to write prescriptions–Figure 10. There is a positive, but weak linear correlation, with r = 0.35817, between the number of patients and GPs.

**Figure 11:**
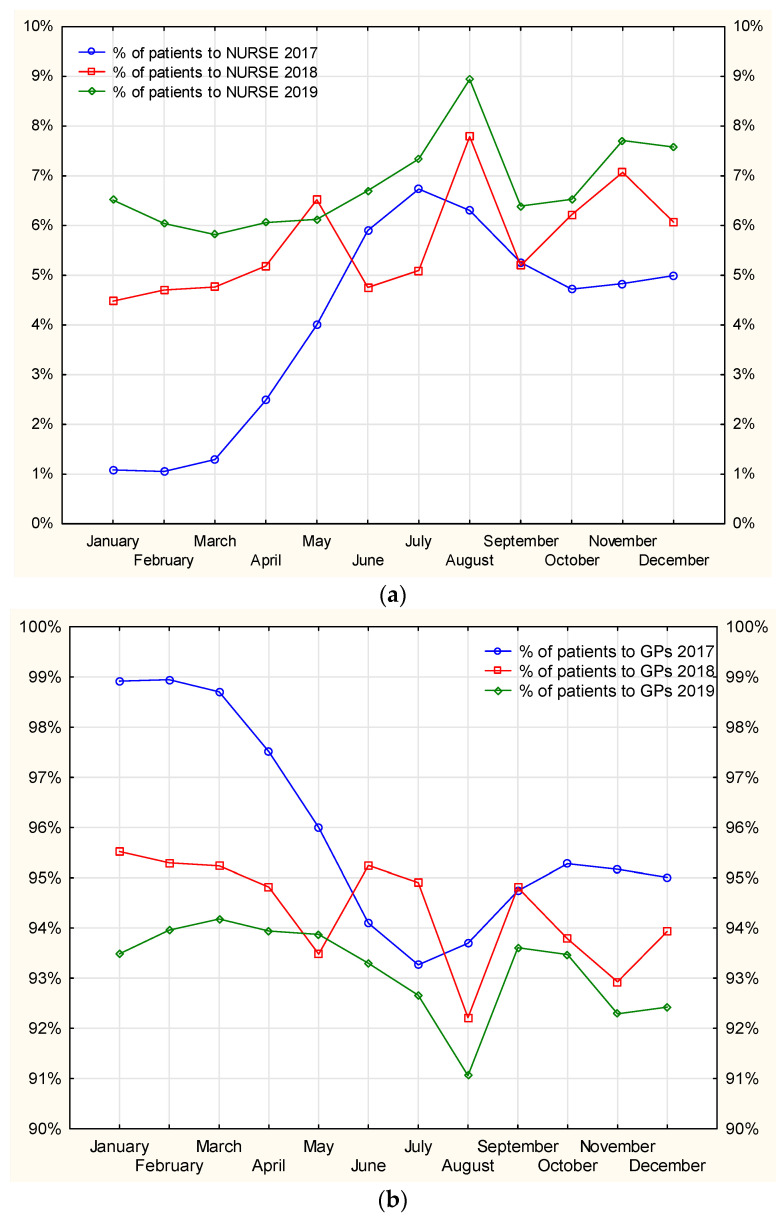
Percentage of patients visiting the nurse (**a**) and GP in each of the analyzed years separately (**b**).

An increasing tendency for prescription visits carried out by nurses, with a simultaneous downward trend in the same type of visits carried out by doctors employed at MDC could be observed–Figure 11. Acute cases are typically handled by doctors, however, during doctors’ annual leave the management of chronic diseases clearly becomes the task of nurses, and their medication prescribing significantly increases. Therefore, patients have no problem obtaining treatment continuation.

**Figure 12:**
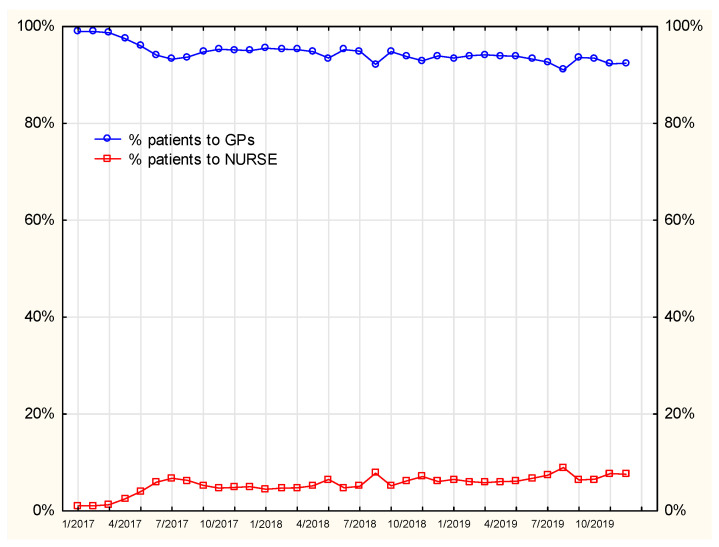
Percentage of patients visiting the GP or the nurse.

In Figure 12, the line graph for the percentage of patients to nurses in July shows three peaks, which also reflect decreases in the number of patients visiting GPs. This indicates the task-shifting, which is of particular importance during holiday seasons when patients would otherwise have a limited access to treatment if it were not for the prescription rights for nurses. Shifting the prescription competences to nurses facilitates pharmacotherapy continuation, thereby maintaining medication accessibility for patients.

**Figure 13:**
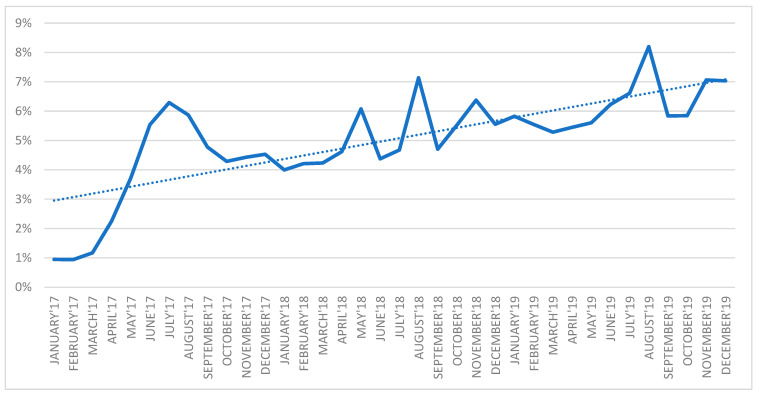
The ratio of prescription visits performed by nurses to all prescription visits.

As Figure 13 shows, the ratio of prescription visits performed by nurses to all the prescription visits systematically increased from 1 percent in January 2017 to 6 percent in December 2019. An increasing number of patients benefited from the opportunity to turn to nurses to continue their therapies.

Regarding the poor availability and quality of health services for medication, particularly where access to doctors can be difficult, the most important solution task-shifting to nurses [39]. According to a Polish study, most of the respondents (55.7 percent) believed that the new competencies would improve accessibility and reduce queues at the doctor [40].

**Figure 14:**
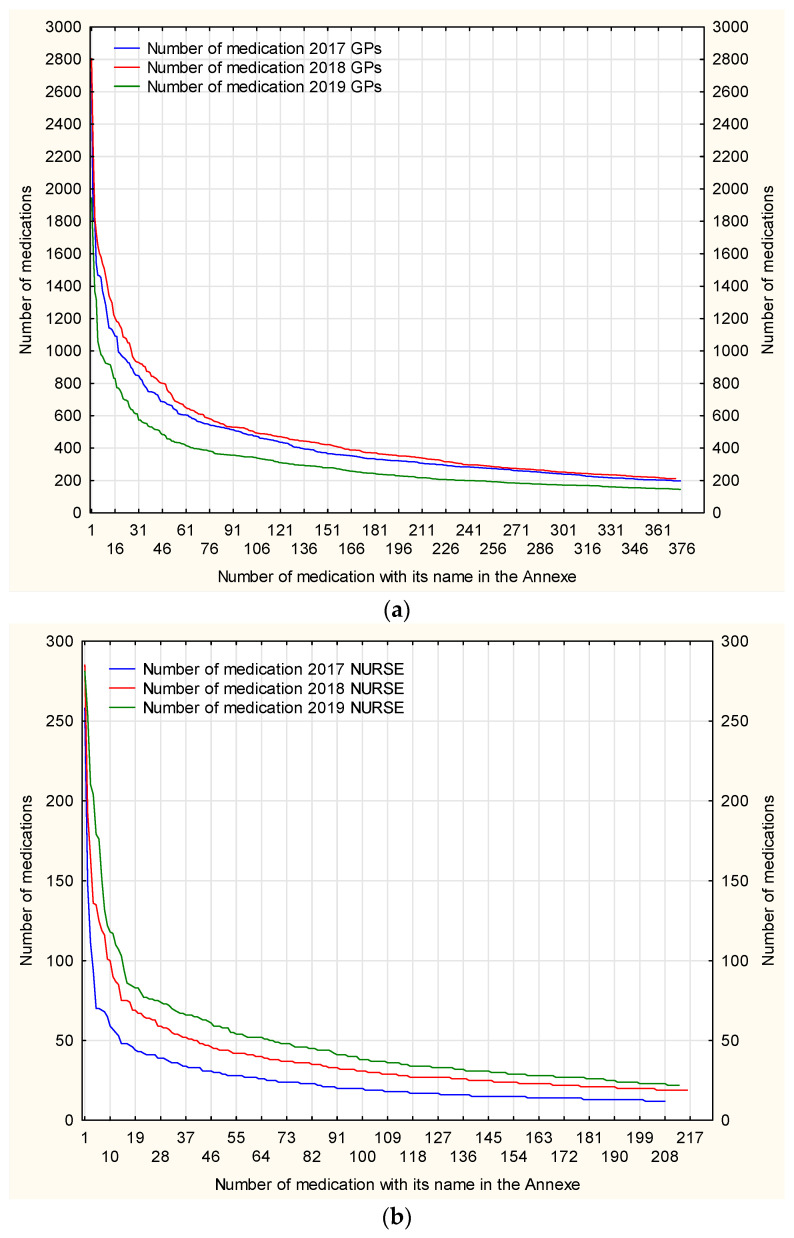
The number of the most common medications prescribed by the nurse (**a**) or GP (**b**) per patient.

According to the data presented in Figure 14, the initial period of task-shifting to nurses (nurse prescribing) was linked to a decreased percentage of medications prescribed by doctors, while a simultaneous rise in the percentage of nurses prescribing medications has been observed since January 2017. The following periods analyzed saw a steady rise in the percentage of medication prescribing by nurses, although the employment rate in this profession did not increase. Furthermore, in July 2017, June and August 2018 and August 2019, a decreased percentage of prescriptions issued by doctors was observed, while the respective values were elevated with regard to nurses.

**Figure 15:**
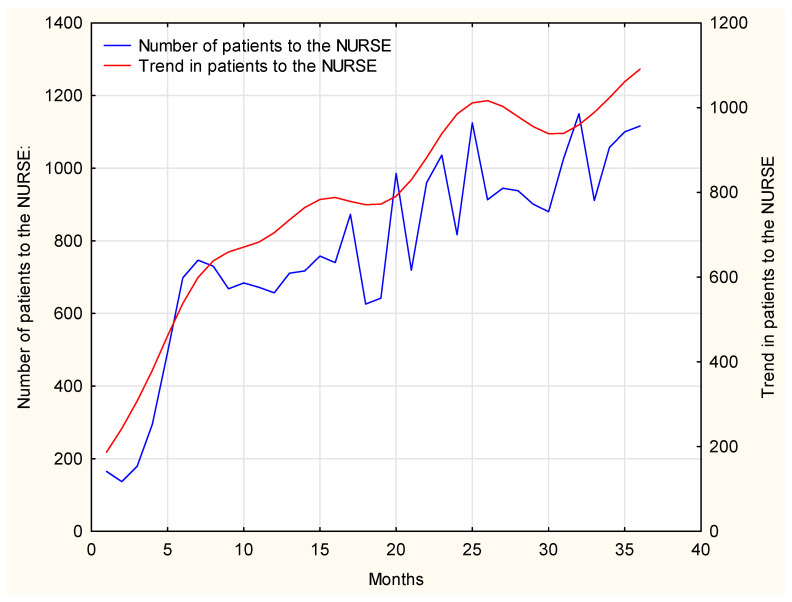
The number of patients visiting the nurse during the 36-month period, with a trend line.

**Figure 16:**
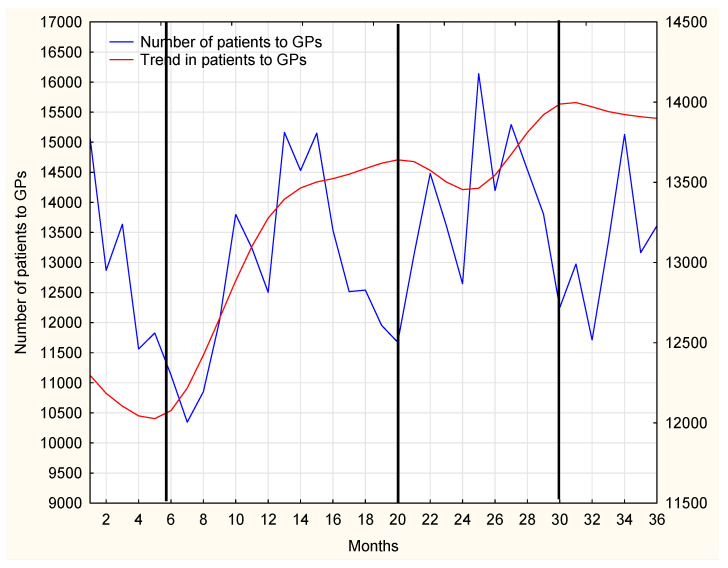
The number of the patients visiting the GP during the 36-month period, with a trend line.

The number of MDC patients has been gradually growing; however, a reduced frequency of prescription visits was recorded. In 2017, the number of prescription visits was markedly elevated in the first months when compared to the number of registered patients. In comparable periods in the following years, the ratio of prescription visits to the increasing number of patients was decreased. Seasonal reduction in the number of prescription visits was registered in holiday months Figure 15 and Figure 16.

## 4. Discussion

Until 2016, patients who needed prescription of medicines to continue their therapies were obliged to provide to PHC a formal document including a list of substances they needed for treatment. Once the medication was prescribed by a doctor, patients could collect the prescription personally or via an attorney or have it delivered by a nurse.

Since 1 January 2016, nurses and midwives have been entitled to issue prescriptions for medications and foods for special medical purposes, referrals to and prescriptions for medical products and referrals for diagnostic tests. Pursuant to Article 15a of the Act of 15 July 2011 on the professions of a nurse and midwife (Journal of Laws (Dz.U.) of 2014, item 1435, as amended) new rights were granted to nurses and midwives with higher university degrees in nursing (BA and MA) and nurses and midwives with a specialist title in nursing or midwifery after completing relevant courses. Candidates recruited for nursing or midwifery departments in the academic year 2016/2017 were educated based on new standards, which included the requirements of new professional qualifications. This meant that they would no longer need to complete specialist courses to that extent, and when graduating from universities they would already have sufficient competences to order and continue treatment as an integral competence acquired after education at both levels of professional training: MA and BA [41].

According to the data from National Health Fund (NHF) obtained between 1 January 2016 and 30 June 2020, 2523 independent nurses and 321 independent midwives applied to the NHF portal for unique prescription identification numbers. Healthcare providers, in turn, registered unique identification numbers for 1857 nurses and 144 midwives who provide public healthcare. By October 2020 nurses issued 1,972,620 and midwives issued 50,012 prescriptions as continuation of doctors’ recommendations, whereas 25,837 prescriptions were issued by nurses and 621 by midwives as independent orders. Nurses and midwives do not only have the right to prescribe medications for patients, but they also exercise the right pro authored/pro familiar. In the first half of 2020, this type of prescription was issued by 12,437 nurses and midwives jointly, where 11,701 were written by nurses and 736 by midwives [42]. The number of nurses and midwives who issue prescriptions for reimbursable items in the provinces is showed on the Table 1.

By means of ‘e-Prescribing’, healthcare providers can enter prescription information into a computer device—tablets, laptops, or desktop computers—and safely transmit the prescription to pharmacies using an application. Research studies suggest that e-prescribing in the USA reduces prescribing errors, increases efficiency, and helps to economize on healthcare costs. ePrescribing system users report benefits in saving time and improving efficiency [43,44].

There has been a positive impact on doctor–nurse communication since the introduction of ePrescribing [45,46]. According to the NHS, electronic prescribing (ePrescribing) systems can help improve the safety and efficiency of healthcare by aiding the choice, prescribing, administration and supply of medicines. The safety and effectiveness of ePrescribing systems depends on all staff groups being actively engaged in their development and use [47].

Although professional education for both physicians and nurses who issue prescriptions is different, research shows that their initial experience in that respect is comparable. Clinical experience and educational support from experienced staff are important and should be promoted for competent development of the prescribers [48].

The unprecedented situation of the pandemic has revolutionized healthcare systems by the introduction of a new type of service, i.e., online consultation. Consequently, it is also crucial to provide a prescription service online.

The available scientific literature reports that nonmedical healthcare providers, including nurses, proved to be as effective as medical specialists in prescribing medications [25,26]. Some authors reported that nurses were effective in prescribing a wide range of medications such as those administered in chronic diseases, in communicable disease treatment, medications for emergency use and for immediate relief [49]. The type of drugs prescribed by nurses in the evaluated entity is shown in the Table S1.

Research also shows that patients are in favor of the expanded nurse competence in terms of the range of prescribed medication, thereby implying their satisfaction with the services provided by nurses [50].

In the available literature, a more prominent role of nurses in healthcare with regards to the prescribing competence acquisition and development is described as the competences worthy of investments [51]. Another study performed in Poland also found that nurse-prescribing was mainly used to treat pain and infections in an elderly patients group. This study has shown that nurses issued prescriptions mainly on the physician’s orders and for the treatment of chronic conditions in elderly patients. Two years after nurse-prescribing legalization, a five-fold increase in the number of nurse prescriptions was observed [52].

Both in Poland and abroad, opinions on the role that nurses play in medication prescribing have been and still are diverse and frequently very cautious, and the number of nurses who feel insufficiently prepared for medication prescribing is elevated [53]. What plays a vital role in the increased share of nurses in prescription services is an adequately designed education program which should take into account the needs of medical communities [54].

Delegating prescription services in chronic diseases treatment to nurses and thereby taking the burden off the physicians as well as increasing prescription accessibility to patients is a common practice in many countries worldwide, however, the scope of the powers is also significantly extended [23,24,50].

An increase in service accessibility by prescribing medication for the purpose of therapy continuation is one of the elements of the comprehensiveness of healthcare in the patients’ residence areas. Another effect-related issue concerning therapies involves the possibility of therapy discontinuation in the case of ineffectiveness of a specific technology application. This is defined as deprescribing, and has become a newly arising area of medication management. Deprescribing involves a withdrawal of medication which may be ordered as a consequence of adverse events or pharmacotherapy ineffectiveness (reactive deprescribing). Proactive deprescribing, in turn, takes place when the doctor and the patient jointly opt for discontinuation of medications because the expected benefits do not outweigh potential risks. The role which nurses may play in this process is also being more and more widely discussed, and may include the support nurses can offer to patients in their informed decisions on their treatments. Education and continuous professional development of medicine-prescribing nurses must allow for the specificity and complexity of treatment for senior populations, which involves careful observation of the experience of patients taking multiple medications, mainly patients suffering from multiple chronic diseases.

The care provided for patients, particularly polypharmacy patients, requires a multidisciplinary approach to treatment, which considers the role of the nurse in receiving feedback on the therapy’s effects in terms of polypharmacy and its side effects [55,56].

## 5. Conclusions

Structural changes in legislation of the nursing profession improve the access to prescription visits for patients under primary healthcare. Such changes also provide the opportunity for physicians and nurses to complement one another in health service. This conclusion has been proven by analysis of the size of the population receiving medical prescription visits made by a physician in a given month, compared to the population receiving the same service provided by both physicians and nurses. The results clearly demonstrate that nurses contributed to the increase in the number of visits. It was also recorded that, along with growing experience in medical prescribing and potentially also the patients becoming more trustful, the number of the prescriptions issued by nurses is on the rise.

Patients will benefit from extended competences of nurses as a result of:

1. Structure of the system: Easier access to continued therapy, particularly for so-called, first-time patients for whom doctors will gain more time to evaluate their health status;
2. Process: patients with acute conditions may receive treatment plans and prescription visits more promptly, as the queue will be shorter thanks to some chronically ill patients using nursing services. Vulnerable patients will benefit from a comprehensive care plan in their residential area, where nurses may not only prescribe medications but also devote more time to explanations and patient’s education as well as ascertaining the patient’s understanding of recommendations and orders or selecting an alternative support for patients by instructing their caregivers/family on caregiving. The expanded practical role of nurses will enable doctors to focus on new medical cases and recommend necessary treatment more swiftly. Nurses, in turn, can provide more complex care by complementing their healthcare with medical prescriptions necessary to be issued for therapy continuation, especially for more vulnerable patients with poor treatment management. Nurses’ increasing experience will lead to an increase in the accessibility of initial diagnostics carried out by nurses and an elevated number of the prescriptions issued as a result. Nurse prescribing of medicines available in patients’ residential areas will facilitate the reduction in polytherapy, as nurses visiting patients at home have an excellent opportunity to control the supply of medications in use and exclude prescriptions for medication which counteract the effects of other medicinal products.

## Figures and Tables

**Table 1:** The number of nurses and midwives who issue prescriptions for reimbursable items in the provinces where the prescriptions were filled.

NHF Division Identification No.	National Health Found (NHF) Data	Nurses	Midwives
01	Dolnośląski	486	24
02	Kujawsko-Pomorski	513	32
03	Lubelski	443	37
04	Lubuski	150	8
05	Łódzki	384	27
06	Małopolski	533	36
07	Mazowiecki	1108	72
08	Opolski	227	15
09	Podkarpacki	300	19
10	Podlaski	398	21
11	Pomorski	525	26
12	Śląski	757	55
13	Świętokrzyski	419	19
14	Warmińsko-Mazurski	267	20
15	Wielkopolski	420	38
16	Zachodniopomorski	345	24
Total		7275	473

Adapted with permission from Ref [42]. 2020 Ministerstwo Zdrowia.

## Data Availability

The data presented in this study are available on request from the corresponding author.

## Supplementary Materials

The following supporting information can be downloaded at: https://www.mdpi.com/article/10.3390/ijerph19010292/s1, Table S1 presents the list of products most frequently prescribed by doctors and nurses at MDC Siedlce. The list also includes nonprescription medications, dietary supplements and non reimburs able drugs, whose accessibility is not limited by prescriptions. These products constituted approximately 10 percent of all the medications prescribed by doctors, while the list of products most frequently prescribed by nurses included only one nonprescription product. Nurses represent a professional group which do not resort to supplements to continue treatment.
