# Supplementary material for: Increased Accessibility to Primary Healthcare Due to Nurse Prescribing of Medicines
Source: Int J Environ Res Public Health. 2021 Dec 28;19(1):292. doi: 10.3390/ijerph19010292 (PMC8751194; doi:10.3390/ijerph19010292)
Supplement: Supplementary file 1 [file ijerph-19-00292-s001.zip › ijerph-1452961-supplementary.pdf]

Table S1. The list of products most frequently prescribed by doctors and nurses at MDC Siedlce.

	**The most common medications in 2019 MD (according to the ABC method)**	**The most common medications in 2019 Nurses (according to the ABC method)**
1	PECTO DRILL, syrup, 200 ml, 5909990498017	BISOCARD, film-coated tablets, 30 tablets, 5909990804511
2	KALIPOZ PROLONGATUM, prolonged-release tablets, 60 tablets , 5909990257539	DIURESIN SR, film-coated prolonged-release tablets, 30 tablets , 5909990975815
3	PECTO DRILL, lozenges, 20 lozenges, 5909990497911	KALIPOZ PROLONGATUM, prolonged-release tablets, 60 tablets , 5909990257539
4	BISOCARD, film-coated tablets, 30 tablets, 5909990804511	NEDAL, tablets, 28 tablets , 5909990642809
5	NEBILET, tablets, 28 tablets , 5909990670185	MILURIT, tablets, 50 tablets, 5909990163212
6	MILURIT, tablets, 50 tablets, 5909990163212	FUROSEMIDUM POLFARMEX, tablets, 30 tablets , 5909990223794
7	NIMESIL, granules for oral suspention, 30 sachet a 2g, 5909991040338	TERTENSIF SR, film-coated prolonged-release tablets, 30 tablets , 5909990738212
8	DICORTINEFF, ear/eye drops, suspension, 5 ml , 5909990221868	TRITACE 5, tablets, 28 tablets, 5909990478316
9	ASECURIN, capsules, 20 capsules, 5902020845140	PRESTARIUM 5 MG, film-coated tablets, 30 tablets, 5909990337774
10	FUROSEMIDUM POLFARMEX, tablets, 30 tablets , 5909990223794	POLPRIL, tablets, 28 tablets, 5909990924646
11	BERODUAL, nebuliser solution, 20 ml , 5909990101917	AMLOZEK, tablets, 30 tablets , 5909990799718
12	LEVOPRONT, syrup, 120 ml , 5909990910823	INDAPEN SR, prolonged-release tablets, 30 tablets , 5909990665907
13	CONTROLOC 20, gastro-resistant tablets, 28 tablets , 5909990478774	METOCARD ZK, prolonged-release tablets, 28 tablets , 5909990697229
14	ERDOMED, capsules, 20 capsules , 5909990041824	GLUCOPHAGE XR, prolonged-release tablets, 60 tablets, 5909990864461
15	VENTOLIN, pressurised inhalation, suspension, 200 metered actuations, 5909990442010	CONTOUR PLUS, test strips, 50 pieces, 5016003763403
16	DIURESIN SR, film-coated prolonged-release tablets, 30 tablets , 5909990975815	CONTROLOC 20, gastro-resistant tablets, 28 tablets , 5909990478774
17	ENTEROL 250, capsules, 10 capsules, 5909990748419	BISOCARD, film-coated tablets, 30 tablets , 5909990673360
18	TERTENSIF SR, film-coated prolonged-release tablets, 30 tablets , 5909990738212	ATORIS, film-coated tablets, 30 tablets , 5909990991914
19	GLIMBAX, mouthwash and gargle, 200 ml, 5909990645022	FUROSEMIDUM POLPHARMA, tablets, 30 tablets, 5909990135028
20	NEDAL, tablets, 28 tablets , 5909990642809	TRITACE 10, tablets, 28 tablets , 5909990916016
21	ERDOMED, capsules, 10 capsules, 5909990041800	ATORVASTEROL, film-coated tablets, 30 tablets, 5909990077939

22	DORETA, film-coated tablets, 60 tablets , 5909990735167	DIAPREL MR, modified-release tablets, 30 tablets, 5909990774746
23	INDAPEN SR, prolonged-release tablets, 30 tablets , 5909990665907	XARELTO, film-coated tablets, 28 tablets, 5909990910724
24	METMIN, nasal spray, suspension, 140 actuations (18 g), 5909991141004	LETROX 50, tablets, 50 tablets, 5909990374014
25	NEUROVIT, film-coated tablets, 20 tablets , 5909990761500	NEBILENIN, tablets, 28 tablets, 5909990689774
26	ACARD, gastro-resistant tablets, 30 tablets , 5909990672516	BISORATIO 5, tablets, 30 tablets , 5909991015015
27	AMOKSIKLAV, film-coated tablets, 14 tablets , 5909990411115	SPIRONOL, tablets, 100 tablets, 5909990110223
28	NEBBUD, nebuliser suspension, 20 ampulesa 2ml, 5909991005696	NEBICARD, tablets, 28 tablets, 5909990685189
29	AMLOZEK, tablets, 30 tablets , 5909990799718	ROSWERA, film-coated tablets, 28 tablets, 5909990895106
30	MYDOCALM FORTE, film-coated tablets, 30 tablets , 5909990228010	DIUVER, tablets, 30 tablets , 5909990422883
31	STRUCTUM, capsules, 60 capsules, 5909991082215	AXTIL, tablets, 30 tablets , 5909990337972
32	POLPRIL, tablets, 28 tablets, 5909990924646	LETROX 100, tablets, 50 tablets, 5909990168910
33	CONTOUR PLUS, test strips, 50 pieces, 5016003763403	LETROX 75, tablets, 50 tablets, 5909991107260
34	ATORIS, film-coated tablets, 30 tablets , 5909990991914	METOCARD, tablets, 30 tablets , 5909990034420
35	SPIRONOL, tablets, 100 tablets, 5909990110223	POLPRIL, capsules hard, 28 capsules , 5909990694631
36	TRITACE 5, tablets, 28 tablets, 5909990478316	IXELL, test strips, 50 pieces, 5908222562632
37	ACCU-CHEK PERFORMA, test strips, 50 pieces, 4015630980987	POLFENON, film-coated tablets, 20 tablets, 5909990034123
38	FANIPOS, nasal spray, suspension, 120 actuations, 5909990570720	MILURIT, tablets, 30 tablets, 5909990414819
39	PRESTARIUM 5 MG, film-coated tablets, 30 tablets, 5909990337774	POLPRIL, tablets, 28 tablets, 5909990924608
40	ARGENTIN - T SPRAY D/GARDŁA, -, 20 ml, 5902768521054	METIZOL, tablets, 50 tablets, 5909990231911
41	SUMAMED, film-coated tablets, 3 tablets, 5909990742417	TORAMIDE, tablets, 30 tablets , 5909991161910
42	CIPRONEX, film-coated tablets, 10 tablets , 5909990334964	LIPANTHYL 267 M, capsules, 30 capsules, 5909990492817
43	ROSWERA, film-coated tablets, 28 tablets, 5909990895106	TORAMIDE, tablets, 30 tablets , 5909991162016
44	KETONAL FORTE, film-coated tablets, 30 tablets, 5909990046485	PRIMACOR, film-coated tablets, 28 tablets , 5909990928521
45	CLATRA, tablets, 30 tablets, 5909990840335	ACCU-CHEK PERFORMA, test strips, 50 pieces, 4015630980987
46	BISORATIO 5, tablets, 30 tablets , 5909991015015	ZAHRON, film-coated tablets, 28 tablets, 5909990802623
47	GLUCOPHAGE XR, prolonged-release tablets, 60 tablets, 5909990864461	ZAHRON, film-coated tablets, 28 tablets, 5909990802562
48	DICLODUO, modified-release capsules, 30 capsules , 5909990752010	DORETA, film-coated tablets, 60 tablets, 5909990735167
49	DUOMOX, tablets, 20 tablets, 5909990063413	POLPRIL, tablets, 28 tablets, 5909990924653
50	NO-SPA FORTE, tablets, 20 tablets, 5909990865321	METFORMAX 500, tablets, 60 tablets, 5909990935253
51	BISOCARD, film-coated tablets, 30 tablets , 5909990673360	AMLOPIN 5 MG, tablets, 30 tablets , 5909990048939

52	DIUVER, tablets, 30 tablets , 5909990422883	DORETA, film-coated tablets, 60 tablets, 5909990936595
53	MILURIT, tablets, 30 tablets, 5909990414819	POLPRAZOL, gastro-resistant capsules, hard, 28 capsules , 5909990772667
54	CONTROLOC 40, gastro-resistant tablets, 28 tablets , 5909990689859	TORVALIPIN, film-coated tablets, 30 tablets , 5909990053230
55	FUROSEMIDUM POLPHARMA, tablets, 30 tablets, 5909990135028	ROMAZIC, film-coated tablets, 30 tablets, 5909990919604
56	FLUIMUCIL, effervescent tablets, 10 tablets, 5909997199122	KETREL, film-coated tablets, 30 tablets , 5909990430840
57	DEVIKAP, oral solution, 10 ml, 5909990260218	METOCARD ZK, prolonged-release tablets, 28 tablets , 5909990697205
58	NASEN, film-coated tablets, 20 tablets , 5909991084431	POLPRIL, capsules hard, 28 capsules , 5909990694655
59	KLABAX, film-coated tablets, 14 tablets , 5909990045532	ATORIS, film-coated tablets, 30 tablets , 5909990991815
60	AMOTAKS, tablets, 16 tablets , 5909990691319	VIVACE 10 MG, tablets, 30 tablets , 5909990610532
61	NEBILENIN, tablets, 28 tablets, 5909990689774	CONCOR COR 2,5, film-coated tablets, 28 tablets , 5909990859016
62	ERDOMED, powder for oral suspension, 100 ml , 5909990052929	NOLIPREL FORTE, film-coated tablets, 30 tablets , 5909990055029
63	KETREL, film-coated tablets, 30 tablets , 5909990430840	EUTHYROX N 75, tablets, 100 tablets , 5909991051327
64	NEBBUD, nebuliser suspension, 20 ampules a 2ml, 5909991005733	ACARD, gastro-resistant tablets, 30 tablets , 5909990672516
65	RUPAFIN 10, tablets, 30 tablets , 5909990083282	SOBYCOR, film-coated tablets, 30 tablets, 5909991097400
66	FLEGAMINA BABY, oral drops, 30 ml, 5909990114214	ROSWERA, film-coated tablets, 28 tablets, 5909990895250
67	ROSWERA, film-coated tablets, 28 tablets, 5909990895250	ACENOCUMAROL WZF, tablets, 60 tablets, 5909990055715
68	ACENOCUMAROL WZF, tablets, 60 tablets, 5909990055715	LIPANTHYL SUPRA 160, film-coated tablets, 30 tablets , 5909990903917
69	BIOFENAC, film-coated tablets, 20 tablets, 5909990881307	XARELTO, film-coated tablets, 28 tablets, 5909990910656
70	CLATRA, tablets, 10 tablets, 5909990840298	POLSART, tablets, 28 tablets, 5909990936700
71	CYCLO 3 FORT, capsules hard, 30 capsules, 5909990884315	ZOFENIL 30, film-coated tablets, 28 tablets, 5909991129439
72	DORETA, film-coated tablets, 60 tablets, 5909990936595	KALIPOZ PROLONGATUM, prolonged-release tablets, 30 tablets , 5909990257515
73	KETONAL, żel, 100 g , 5909990634033	EUTHYROX N 50, tablets, 100 tablets , 5909991051228
74	BACLOFEN POLPHARMA, tablets, 50 tablets, 5909990033713	TRITACE 2,5, tablets, 28 tablets , 5909990478217
75	DIPHERGAN, tablets drażowane, 20 tablets, 5909990228317	ACTELSAR, tablets, 28 tablets, 5909990891863
76	IBUPROFEN -PABI, tablets drażowane, 60 tablets , 5909990132928	AXTIL, tablets, 30 tablets , 5909990337989
77	ZINNAT, film-coated tablets, 14 tablets, 5909990083435	NIMESIL, granules for oral suspention, 30 sachet a 2g, 5909991040338
78	OPOKAN-KETO, żel, 100 g , 5906071004051	CONTROLOC 40, gastro-resistant tablets, 28 tablets , 5909990689859
79	TRITACE 10, tablets, 28 tablets , 5909990916016	EBIVOL, tablets, 30 tablets , 5909990662425
80	XARELTO, film-coated tablets, 28 tablets, 5909990910724	SIMVASTEROL, film-coated tablets, 28 tablets , 5909990927715
81	ZAHRON, film-coated tablets, 28 tablets, 5909990802623	ROSWERA, film-coated tablets, 28 tablets, 5909990895380

82	KALIPOZ PROLONGATUM, prolonged-release tablets, 30 tablets , 5909990257515	PRESTARIUM 10 MG, film-coated tablets, 30 tablets, 5909990336081
83	HITAXA, orodispersible tablets, 30 tablets, 5909990981373	AVEDOL, film-coated tablets, 30 tablets , 5909990074051
84	LETROX 100, tablets, 50 tablets, 5909990168910	TRIMEDUCTAN MR, film-coated modified-release tablets, 60 tablets , 5909991151812
85	TORAMIDE, tablets, 30 tablets , 5909991162016	KETONAL FORTE, film-coated tablets, 30 tablets, 5909990046485
86	OLFEN 75 SR, film-coated prolonged-release tablets, 30 tablets , 5909990974122	AMLOZEK, tablets, 30 tablets , 5909990799817
87	TORAMIDE, tablets, 30 tablets , 5909991161910	IPP 20, gastro-resistant tablets, 28 tablets , 5909990085033
88	MILGAMMA N, solution for injection, 5 ampules, 5909991020415	ATORVOX, film-coated tablets, 30 tablets , 5909990573530
89	METOCARD, tablets, 30 tablets , 5909990034420	VENTOLIN, pressurised inhalation, suspension, 200 metered actuations, 5909990442010
90	PIMAFUCORT, cream, 15 g , 5909990191710	PREDUCTAL MR, modified-release tablets, 60 tablets , 5909990846115
91	BUDERHIN, nasal spray, suspension, 1 poj.a 200actuations, 5909990332311	EUTHYROX N 75, tablets, 50 tablets, 5909991051310
92	POLPRAZOL, gastro-resistant capsules, hard, 28 capsules , 5909990772667	CAPTOPRIL, tablets, 30 tablets, 5909990044030
93	POLFENON, film-coated tablets, 20 tablets, 5909990034123	CONCOR COR 1,25, film-coated tablets, 28 tablets , 5909990858910
94	POLPRIL, tablets, 28 tablets, 5909990924608	DIAPREL MR, modified-release tablets, 60 tablets , 5909990443017
95	IPP 20, gastro-resistant tablets, 28 tablets , 5909990085033	SPIRONOL, tablets, 20 tablets (1x20), 5909990110216
96	LETROX 75, tablets, 50 tablets, 5909991107260	EFFOX LONG 50, prolonged-release tablets, 30 tablets, 5909990368624
97	ATORVASTEROL, film-coated tablets, 30 tablets, 5909990077939	ZARANTA, film-coated tablets, 28 tablets , 5909990777785
98	DIAPREL MR, modified-release tablets, 30 tablets, 5909990774746	INDIX COMBI, film-coated tablets, 30 tablets, 5909991050344
99	CONTIX, gastro-resistant tablets, 14 tablets , 5909991128814	LECALPIN, film-coated tablets, 30 tablets, 5909990774555
100	DEXILANT, modified-release capsules hard, 28 capsules, 5909991096748	INDIX SR, prolonged-release tablets, 30 tablets , 5909991025014
101	BIOTYK, capsules, 10 capsules	NOLPAZA 20, gastro-resistant tablets, 90 tablets, 5909990845521
102	HITAXA, oral solution, 150 ml , 5909990981458	INS. NOVORAPID PENFILL, solution for injection, 5 wkł.a 3ml, 5909990451814
103	METOCARD ZK, prolonged-release tablets, 28 tablets , 5909990697229	ATROX 20, film-coated tablets, 30 tablets , 5909991124717
104	LETROX 50, tablets, 50 tablets, 5909990374014	VIVACE 5 MG, tablets, 30 tablets , 5909990610495
105	TELFEXO 180, film-coated tablets, 20 tablets , 5909990081837	FINASTER, film-coated tablets, 30 tablets , 5909991151218

106	KALDYUM, prolonged-release capsules hard, 100 capsules, 5909990822126	TRIFAS 10, tablets, 30 tablets, 5909990471324
107	TRIDERM, ointment, 15 g , 5909990401925	SETAL MR, modified-release tablets, 60 tablets , 5909990646746
108	CAPTOPRIL JELFA, tablets, 30 tablets , 5909990673384	BETO 50 ZK, prolonged-release tablets, 28 tablets , 5909990220809
109	TAROMENTIN, film-coated tablets, 14 tablets , 5909991087715	METFORMAX 500, tablets, 30 tablets , 5909990126316
110	RAMOCLAV, film-coated tablets, 14 tablets, 5909991042073	ATORIS, film-coated tablets, 30 tablets, 5909990623464
111	AXTIL, tablets, 30 tablets , 5909990337972	GLUCOPHAGE 500 MG, film-coated tablets, 30 tablets, 5909990789276
112	LACIDAR, tablets, 20 tablets, 5907670697033	MESOPRAL, gastro-resistant capsules, hard, 28 capsules , 5909990876280
113	FURAGINUM TEVA, tablets, 30 tablets, 5909990988235	NONPRES, film-coated tablets, 30 tablets, 5909991014728
114	DICLOBERL RETARD, prolonged-release capsules, 20 capsules, 5909990492213	LIPANTHYL SUPRA 215, film-coated tablets, 30 tablets , 5909990431342
115	NOLPAZA 20, gastro-resistant tablets, 28 tablets , 5909990075003	BETALOC ZOK 50, prolonged-release tablets, 28 tablets , 5909990738632
116	ATORIS, film-coated tablets, 30 tablets , 5909990991815	NOLPAZA 20, gastro-resistant tablets, 56 tablets , 5909990075041
117	MESOPRAL, gastro-resistant capsules, hard, 28 capsules , 5909990876280	STRUCTUM, capsules, 60 capsules, 5909991082215
118	POLPRIL, capsules hard, 28 capsules , 5909990694631	EUTHYROX N 25, tablets, 100 tablets , 5909991051129
119	MACROMAX, film-coated tablets, 3 tablets , 5909990713608	RANIGAST, film-coated tablets, 60 tablets , 5909990206728
120	NEBBUD (NEBUCORT), nebuliser suspension, 10 ampules a 2ml, 5909991228859	RANLOSIN, prolonged-release capsules, 30 capsules, 5909990048007
121	ZAHRON, film-coated tablets, 28 tablets, 5909990802562	LOKREN 20, film-coated tablets, 28 tablets , 5909990037414
122	CONTIX, gastro-resistant tablets, 28 tablets , 5909991128838	VALSACOR 80, film-coated tablets, 28 tablets , 5909990074945
123	MUCOSOLVAN INHALACJE, nebuliser solution, 100 ml, 5909990102518	SIOFOR 1000, film-coated tablets, 90 tablets, 5909990221004
124	MILGAMMA 100, coated tablets, 30 tablets, 5909991080419	ZAHRON, film-coated tablets, 28 tablets, 5909990802685
125	ZYRTEC, oral drops, solution, 20 ml, 5909990184736	BISOCARD, film-coated tablets, 60 tablets, 5909990804528
126	RANIGAST, film-coated tablets, 60 tablets , 5909990206728	TAMISPRAS, prolonged-release tablets, 30 tablets, 5909990980451
127	DEXILANT, modified-release capsules hard, 28 capsules, 5909991096762	ACTELSAR HCT, tablets, 28 tablets, 5909991056773
128	FLOXAL, eye drops, solution, 5 ml , 5909990411610	DIAGNOSTIC GOLD STRIP, test strips, 50 pieces, 5906881862681
129	AUGMENTIN, film-coated tablets, 14 tablets , 5909990717521	TULIP, film-coated tablets, 30 tablets , 5909990998913
130	POLPRIL, tablets, 28 tablets, 5909990924653	VALSACOR 160, film-coated tablets, 28 tablets , 5909990074969
131	TRILAC, capsules hard, 20 capsules, 5909990701919	NITRENDYPINA EGIS, tablets, 60 tablets, 5909990694785

132	PRAMOLAN, film-coated tablets, 20 tablets, 5909990205813	ACCU-CHEK ACTIVE, test strips, 50 pieces, 4015630056316
133	POLTRAM COMBO, film-coated tablets, 60 tablets, 5909990841004	KALDYUM, prolonged-release capsules hard, 50 capsules, 5909990822119
134	TRITTICO CR, prolonged-release tablets, 30 tablets , 5909990918621	BIOSOTAL 40, tablets, 60 tablets , 5909990365715
135	EMANERA, gastro-resistant capsules, hard, 28 capsules, 5909990926497	BIBLOC, film-coated tablets, 30 tablets, 5909990694105
136	OSPAMOX, film-coated tablets, 16 tablets, 5909990293322	AMLOPIN 10 MG, tablets, 30 tablets , 5909990048977
137	DIGOXIN TEVA, tablets, 30 tablets , 5909990012114	BIOPRAZOL, capsules hard, 28 capsules, 5909990880225
138	ACARD, gastro-resistant tablets, 60 tablets , 5909990672523	MESOPRAL, gastro-resistant capsules, hard, 28 capsules, 5909990876778
139	XARELTO, film-coated tablets, 28 tablets, 5909990910656	LACIPIL, film-coated tablets, 28 tablets , 5909990650620
140	IXELL, test strips, 50 pieces, 5908222562632	METFORMAX 850, tablets, 60 tablets, 5909990935260
141	METFORMAX 500, tablets, 60 tablets, 5909990935253	EUTHYROX N 88 MCG, tablets, 50 tablets, 5909990718986
142	SINUPRET, coated tablets, 50 tablets, 5909990763818	PRENESSA, tablets, 30 tablets , 5909990569311
143	LIPANTHYL SUPRA 160, film-coated tablets, 30 tablets , 5909990903917	WARFIN, tablets, 100 tablets, 5909990622382
144	CONCOR COR 2,5, film-coated tablets, 28 tablets , 5909990859016	HYDROXYZINUM VP, film-coated tablets, 30 tablets , 5909990188819
145	NEBICARD, tablets, 28 tablets, 5909990685189	NOLPAZA 20, gastro-resistant tablets, 28 tablets , 5909990075003
146	BIORACEF, film-coated tablets, 14 tablets , 5909990063703	AXTIL, tablets, 30 tablets , 5909990337958
147	ZYRTEC, film-coated tablets, 30 tablets , 5909990184637	ACTELSAR HCT, tablets, 28 tablets, 5909991056247
148	SOBYCOR, film-coated tablets, 30 tablets, 5909991097400	VESSEL DUE F, soft capsules, 50 capsules, 5909990039616
149	LACIPIL, film-coated tablets, 28 tablets , 5909990650620	GLIBETIC 4MG, tablets, 30 tablets , 5909991097318
150	METIZOL, tablets, 50 tablets, 5909990231911	TRIFAS COR, tablets, 30 tablets, 5909990471225
151	AXTIL, tablets, 30 tablets , 5909990337989	AVEDOL, film-coated tablets, 30 tablets , 5909990074099
152	POLSART, tablets, 28 tablets, 5909990936700	HEPAREGEN, tablets, 100 tablets, 5909990029327
153	TOLPERIS VP, film-coated tablets, 30 tablets , 5909990959716	BETALOC ZOK 25, prolonged-release tablets, 28 tablets , 5909990916818
154	DICOFLOR 60, capsules, 10 capsules, 8033300190043	TELMIZEK, tablets, 28 tablets, 5909990902095
155	LIPANTHYL SUPRA 215, film-coated tablets, 30 tablets , 5909990431342	EUTHYROX N 50, tablets, 50 tablets, 5909991051211
156	AULIN, granules for oral suspension, 30 sachets a 2g, 5909990411436	CLATRA, tablets, 30 tablets, 5909990840335
157	MESOPRAL, gastro-resistant capsules, hard, 28 capsules , 5909990876778	POLSART, tablets, 28 tablets, 5909990936670
158	EBILFUMIN, capsules hard, 10 capsules	TIALORID MITE, tablets, 50 tablets, 5909990373819
159	ROMAZIC, film-coated tablets, 30 tablets, 5909990919604	POLTRAM COMBO, film-coated tablets, 60 tablets, 5909990841004
160	AUGMENTIN, film-coated tablets, 14 tablets, 5909997198385	EUTHYROX N 100, tablets, 100 tablets , 5909991051426
161	CAPTOPRIL, tablets, 30 tablets, 5909991160913	ATORVASTEROL, film-coated tablets, 30 tablets, 5909990077847

162	IBUPROM ZATOKI, film-coated tablets, 12 tablets , 5909990965021	BETO 50 ZK, prolonged-release tablets, 30 tablets, 5909997198354
163	SIMVASTEROL, film-coated tablets, 28 tablets , 5909990927715	CONTIX, gastro-resistant tablets, 28 tablets , 5909991128838
164	DEFLEGMIN, prolonged-release capsules, 10 capsules , 5909990369218	ALDAN, tablets, 30 tablets , 5909991008635
165	SIRDALUD, tablets, 30 tablets, 5909990671311	VICEBROL FORTE, tablets, 90 tablets, 5909990692491
166	ZINNAT, granules for oral suspension, 50 ml (39,98 g granulatu), 5909990468812	TOLURA, tablets, 28 tablets , 5909997077673
167	LEVOPRONT, syrup, 200 ml , 5909990669905	CAVINTON FORTE, tablets, 90 tablets, 5909991028923
168	AZYCYNA, film-coated tablets, 3 tablets, 5909991098520	DIUVER, tablets, 30 tablets , 5909991003821
169	METOCARD ZK, prolonged-release tablets, 28 tablets , 5909990697205	ACTELSAR, tablets, 28 tablets, 5909990891832
170	DALACIN C, capsules, 16 capsules , 5909990306435	PROTEVASC SR, film-coated prolonged-release tablets, 60 tablets, 5909990919680
171	PIMAFUCORT, ointment, 15 g , 5909990210015	EMANERA, gastro-resistant capsules, hard, 28 capsules, 5909990926497
172	INDIX SR, prolonged-release tablets, 30 tablets , 5909991025014	NONPRES, film-coated tablets, 30 tablets, 5909991014759
173	HYDROXYZINUM VP, film-coated tablets, 30 tablets , 5909990188819	EUTHYROX N 25, tablets, 50 tablets, 5909991051112
174	POLTRAM COMBO, film-coated tablets, 30 tablets, 5909990840991	ACARD, gastro-resistant tablets, 60 tablets , 5909990672523
175	AMOKSIKLAV, powder for oral suspension, 70 ml (17,5 g), 5909990894826	ATORVASTEROL, film-coated tablets, 30 tablets, 5909990078028
176	NOLPAZA 20, gastro-resistant tablets, 90 tablets, 5909990845521	AREPLEX, film-coated tablets, 28 tablets , 5909991167011
177	SPIRONOL, tablets, 20 tablets (1x20), 5909990110216	JARDIANCE, film-coated tablets, 30 tablets , 5909991138516
178	NEBBUD, nebuliser suspension, 10 ampulesa 2ml, 5909991228842	KALDYUM, prolonged-release capsules hard, 100 capsules, 5909990822126
179	EUTHYROX N 50, tablets, 100 tablets , 5909991051228	NOLIPREL BI-FORTE, film-coated tablets, 30 tablets, 5909990707782
180	TRITACE 2,5, tablets, 28 tablets , 5909990478217	EUTHYROX N 125, tablets, 100 tablets , 5909991051525
181	ACLEXA, capsules hard, 30 capsules, 5909991108373	ESPIRO, film-coated tablets, 30 tablets, 5909990971473
182	AMLOZEK, tablets, 30 tablets , 5909990799817	DIURED, tablets, 30 tablets , 5909990734504
183	SORBIFER DURULES, prolonged-release tablets, 50 tablets, 5909990864553	TORAMIDE, tablets, 30 tablets , 5909991162115
184	NEUROVIT, film-coated tablets, 100 tablets, 5909990761531	HITAXA, orodispersible tablets, 30 tablets, 5909990981373
185	HYDROXYZINUM VP, film-coated tablets, 30 tablets, 5909990188710	ABRA, test strips, 50 szt., 5907581253625
186	EMANERA, gastro-resistant capsules, hard, 28 capsules, 5909990926534	ROMAZIC, film-coated tablets, 30 tablets, 5909990919659
187	PREDUCTAL MR, modified-release tablets, 60 tablets , 5909990846115	BIBLOC, film-coated tablets, 30 tablets, 5909990694167
188	CITRAFLEET, powder for oral solution, 2 sachets, 5909991076245	LERCAN, film-coated tablets, 28 tablets, 5391519921098
189	ENTEROMAX, capsules, 10 capsules, 5907698302780	PRADAXA, capsules hard, 60 capsules , 5909990887484

190	AMLOPIN 5 MG, tablets, 30 tablets , 5909990048939	NITRENDYPINA EGIS, tablets, 60 tablets, 5909990694754
191	ATROVENT N, pressurised inhalation, solution, 10 ml (200 actuations), 5909990999019	TRITTICO CR, prolonged-release tablets, 30 tablets , 5909990918621
192	FERVEX, granules for oral solution, 8 sachets, 5909990363117	DEVIKAP, oral solution, 10 ml, 5909990260218
193	FURAGINUM ADAMED, tablets, 30 tablets, 5909990357215	TOLURA, tablets, 28 tablets , 5909997077604
194	CERUTIN, film-coated tablets, 100 tablets , 5909990882823	COSOPT, eye drops, solution, 1 but.a 5ml, 5909990442423
195	GARDVIT A+E SPRAY NA SUCHE GARDŁO, spray, 30 ml, 5901968037013	SIOFOR 850, film-coated tablets, 120 tablets, 5909990457335
196	PRIMACOR, film-coated tablets, 28 tablets , 5909990928521	AMLOPIN, tablets, 30 tablets, 5909991337742
197	EUTHYROX N 75, tablets, 100 tablets , 5909991051327	POLSART PLUS, tablets, 28 tablets, 5909991079598
198	HEPAREGEN, tablets, 100 tablets, 5909990029327	MEMOTROPIL, film-coated tablets, 60 tablets , 5909990873746
199	POLPRIL, capsules hard, 28 capsules , 5909990694655	DICLOBERL RETARD, prolonged-release capsules, 20 capsules, 5909990492213
200	MOMESTER, nasal spray, suspension, 1 but.a 140 actuations (18 g), 5909991195366	METFORMAX SR 500, prolonged-release tablets, 60 tablets, 5909990933167
201	NALGESIN FORTE, film-coated tablets, 30 tablets, 5909991023805	ROMAZIC, film-coated tablets, 30 tablets, 5909990919574
202	OLFEN UNO, modified-release tablets, 30 tablets , 5909990457120	GLUCOPHAGE XR, prolonged-release tablets, 60 tablets, 5909990213436
203	SKUDEXA, film-coated tablets, 20 tablets, 5909991274580	TELMIZEK, tablets, 28 tablets, 5909990902002
204	PRAMOLAN, film-coated tablets, 56 tablets, 5909991228439	DEPAKINE CHRONO 500, film-coated prolonged-release tablets, 30 tablets , 5909990694426
205	METFORMAX SR 500, prolonged-release tablets, 30 tablets , 5909990652112	HYDROXYZINUM VP, film-coated tablets, 30 tablets, 5909990188710
206	BUTAPIRAZOL, ointment, 30 g , 5909990142910	CONTOUR TS, test strips, 50 pieces, 5016003183904
207	AGLAN 15, tablets, 30 tablets, 5909990610006	GLUCOPHAGE XR, prolonged-release tablets, 30 tablets , 5909990213429
208	LOKREN 20, film-coated tablets, 28 tablets , 5909990037414	BIOSOTAL 80, tablets, 30 tablets , 5909990365616
209	POLSART, tablets, 28 tablets, 5909990936670	BISOCARD, film-coated tablets, 30 tablets, 5909990804412
210	LIPANTHYL 267 M, capsules, 30 capsules, 5909990492817	ZOCOR 20, film-coated tablets, 28 tablets , 5909990366026
211	FINASTER, film-coated tablets, 30 tablets , 5909991151218	RUPAFIN, oral solution, 120 ml , 5909990991921
212	NASONEX, nasal spray, suspension, 18 g (140 actuations), 5909990732395	DOXANORM, tablets, 30 tablets , 5909990854516
213	CEROXIM, film-coated tablets, 14 tablets, 5909991263409	
214	MIFLONIDE BREEZHALER (MIFLONIDE), inhalation powder, hard capsules, 60 capsules (+ inh.), 5909990926213	

215	BIODACYNA OPHTHALMICUM 0,3%, eye drops, solution, 5 ml, 5909990129317	
216	SETAL MR, modified-release tablets, 60 tablets , 5909990646746	
217	TIALORID MITE, tablets, 50 tablets, 5909990373819	
218	NITRENDYPINA EGIS, tablets, 60 tablets, 5909990694785	
219	EUTHYROX N 75, tablets, 50 tablets, 5909991051310	
220	ATORIS, film-coated tablets, 30 tablets, 5909990623464	
221	KETOPROFENUM FASTUM, żel, 100 g , 5909990569229	
222	NASOMETIN, nasal spray, suspension, 1 but.a 140actuations (18 g), 5909991031275	
223	DIUVER, tablets, 30 tablets , 5909991003821	
224	AMLOPIN, tablets, 30 tablets, 5909991337742	
225	NUROFEN, film-coated tablets, 12 tablets , 5909990706822	
226	ROSWERA, film-coated tablets, 28 tablets, 5909990895380	
227	ATROX 20, film-coated tablets, 30 tablets , 5909991124717	
228	MYDOCALM, film-coated tablets, 30 tablets , 5909990227914	
229	ELOCOM, ointment, 15 g, 5909990278916	
230	BUDIXON NEB, nebuliser suspension, 10 poj.a 2ml, 5906414002782	
231	VALSACOR 160, film-coated tablets, 28 tablets , 5909990074969	
232	EBIVOL, tablets, 30 tablets , 5909990662425	
233	MOVALIS, tablets, 20 tablets, 5909990464418	
234	DIAPREL MR, modified-release tablets, 60 tablets , 5909990443017	
235	BIBLOC, film-coated tablets, 30 tablets, 5909990694105	
236	OROFAR, tablets do ssania, 24 tablets, 5909990933112	
237	EUTHYROX N 25, tablets, 50 tablets, 5909991051112	
238	GLUCOPHAGE XR, prolonged-release tablets, 30 tablets , 5909990213429	
239	HEVIRAN, film-coated tablets, 30 tablets , 5909990840229	
240	LAKCID FORTE, capsules hard, 10 capsules, 5909991067830	
241	BETALOC ZOK 25, prolonged-release tablets, 28 tablets , 5909990916818	

242	MIFLONIDE BREEZHALER (MIFLONIDE), inhalation powder, hard capsules, 60 capsules (+ inh.), 5909990926312	
243	VITAMINUM B 12 WZF, solution for injection, 5 ampulesa 2ml, 5909990244010	
244	APO-NAPRO, tablets, 30 tablets , 5909990661442	
245	TULIP, film-coated tablets, 30 tablets , 5909990998913	
246	VALSACOR 80, film-coated tablets, 28 tablets , 5909990074945	
247	UPROX, modified-release capsules hard, 30 capsules, 5909990566068	
248	FOSTEX, pressurised inhalation, solution, 180 actuations, 5909990054152	
249	ZOFENIL 30, film-coated tablets, 28 tablets, 5909991129439	
250	EFFERALGAN CODEINE, tablets musujące, 16 tablets , 5909990678020	
251	KALDYUM, prolonged-release capsules hard, 50 capsules, 5909990822119	
252	BI-PROFENID, modified-release tablets, 20 tablets , 5909990412112	
253	RUTINACEA COMPLETE, tablets, 30 tablets, 5902020845201	
254	EUTHYROX N 50, tablets, 50 tablets, 5909991051211	
255	EFFOX LONG 50, prolonged-release tablets, 30 tablets, 5909990368624	
256	GLUCOPHAGE 500 MG, film-coated tablets, 30 tablets, 5909990789276	
257	GLUCOPHAGE XR, prolonged-release tablets, 30 tablets , 5909990624751	
258	AULIN, tablets, 30 tablets , 5909990411337	
259	ACCU-CHEK ACTIVE, test strips, 50 pieces, 4015630056316	
260	TRIFAS 10, tablets, 30 tablets, 5909990471324	
261	LOPERAMID WZF, tablets, 30 tablets, 5909990038220	
262	TORAMIDE, tablets, 30 tablets , 5909991162115	
263	MUCOFLUID, nasal spray, solution, 12,5 ml, 5909990184415	
264	TARDYFERON-FOL, film-coated modified-release tablets, 30 tablets, 5909990668816	
265	DORETA, film-coated tablets, 90 tablets, 5909991143923	
266	CONTROLOC 20, gastro-resistant tablets, 14 tablets , 5909990478767	
267	ABRA, test strips, 50 szt., 5907581253625	

268	CEROXIM, film-coated tablets, 20 tablets, 5909991263416	
269	POLOCARD, film-coated gastro-resistant tablets, 60 tablets , 5909990654987	
270	ATORIS, film-coated tablets, 90 tablets, 5909990419173	
271	ZARANTA, film-coated tablets, 28 tablets , 5909990777785	
272	ALVESCO 160, pressurised inhalation, solution, 120 actuation, 5909990212064	
273	POLPRAZOL PPH, gastro-resistant capsules, hard, 28 capsules , 5909990077731	
274	BETALOC ZOK 50, prolonged-release tablets, 28 tablets , 5909990738632	
275	ACIDUM FOLICUM RICHTER, tablets, 30 tablets, 5909990109319	
276	CONCOR COR 1,25, film-coated tablets, 28 tablets , 5909990858910	
277	BELLERGOT, tablets drażowane, 30 tablets , 5909990336418	
278	PANPRAZOX, gastro-resistant tablets, 28 tablets, 5909990817184	
279	RANLOSIN, prolonged-release capsules, 30 capsules, 5909990048007	
280	CAVINTON FORTE, tablets, 90 tablets, 5909991028923	
281	PROKIT, film-coated tablets, 40 tablets, 5909991240578	
282	CAPTOPRIL, tablets, 30 tablets, 5909990044030	
283	WARFIN, tablets, 100 tablets, 5909990622382	
284	THIOCODIN, tablets, 10 tablets, 5909990240012	
285	POLVERTIC, tablets, 60 tablets , 5909990646036	
286	TRIFAS COR, tablets, 30 tablets, 5909990471225	
287	LORATADYNA GALENA, tablets, 30 tablets , 5909990795420	
288	DICLODUO COMBI, modified-release hard capsules, 30 capsules , 5909991220594	
289	BUDIXON NEB, nebuliser suspension, 20 x 2ml, 5906414002522	
290	MONURAL, granules for oral solution, 1 sachets a 8g, 5909990699117	
291	EUTHYROX N 25, tablets, 100 tablets , 5909991051129	
292	SPASMOLINA, capsules hard , 20 capsules, 5909990456314	
293	ARGOSULFAN, cream, 40 g, 5909990237319	
294	LIPOMAL, syrup, 125 g, 5909991034719	

295	METOCLOPRAMIDUM POLPHARMA, tablets, 50 tablets , 5909990084722	
296	GROPRINOSIN, syrup, 150 ml, 5909991095697	
297	MULTILAC, capsules, 10 capsules , 5908222562595	
298	TAMISPRAS, prolonged-release tablets, 30 tablets, 5909990980451	
299	PRESTARIUM 10 MG, film-coated tablets, 30 tablets, 5909990336081	
300	NASOMETIN, nasal spray, suspension, 1 but.a 140actuations (18 g), 5909991309206	
301	VICEBROL, tablets, 100 tablets, 5909990223305	
302	SUMAMED FORTE, powder for oral suspension, 20 ml , 5909990742219	
303	TROSICAM, orodispersible tablets, 20 tablets, 5909990866625	
304	NYSTATYNA TEVA, granules for oral suspension, 24 ml, 5909990231515	
305	TELMIZEK, tablets, 28 tablets, 5909990902095	
306	TOBREX, eye drops, solution, 5 ml, 5909990189410	
307	syrup PRAWOŚLAZOWY ALTE, -, 125 g, 5909990024469	
308	AZYCYNA, film-coated tablets, 6 tablets, 5909991098421	
309	NOLIPREL FORTE, film-coated tablets, 30 tablets , 5909990055029	
310	TASECTAN, sachets, 20 sachets, 3830044941048	
311	ACC 200, tablets musujące, 20 tablets, 5909990331314	
312	ZAHRON, film-coated tablets, 28 tablets, 5909990802685	
313	OSPAMOX 1000 MG, film-coated tablets, 20 tablets, 5909990778041	
314	FASTUM, żel, 60 g, 5909997199979	
315	BIOPRAZOL, capsules hard, 28 capsules, 5909990880225	
316	DETREOMYCYNA 2%, ointment, 5 g , 5909990328611	
317	ENTEROL 250, capsules, 20 capsules, 5909990748426	
318	RIDLIP, film-coated tablets, 28 tablets, 5909991000141	
319	DIAGNOSTIC GOLD STRIP, test strips, 50 pieces, 5906881862681	
320	ALERPROF, tablets, 30 tablets , 5909991351939	
321	AVEDOL, film-coated tablets, 30 tablets , 5909990074051	
322	GLUCOPHAGE XR, prolonged-release tablets, 60 tablets, 5909990213436	

323	AUGMENTIN, powder for oral suspension, 70 ml (10,6 g), 5909990419326	
324	AMOTAKS DIS, tablets, 16 tablets , 5909991043728	
325	POLOCARD, film-coated gastro-resistant tablets, 30 tablets , 5909990654970	
326	BISOCARD, film-coated tablets, 60 tablets, 5909990804528	
327	BETO 50 ZK, prolonged-release tablets, 28 tablets , 5909990220809	
328	FINASTER, film-coated tablets, 90 tablets , 5909990811045	
329	NYSTATYNA TEVA, granules for oral suspension, 28 ml , 5909991307240	
330	INDIX COMBI, film-coated tablets, 30 tablets, 5909991050344	
331	ATORVASTEROL, film-coated tablets, 30 tablets, 5909990077847	
332	HYDROXYZINUM ADAMED, film-coated tablets, 30 tablets, 5909991278892	
333	ASPARGIN, tablets, 50 tablets, 5909990210718	
334	FROMILID UNO, modified-release tablets, 5 tablets, 5909991109813	
335	ROMAZIC, film-coated tablets, 30 tablets, 5909990919574	
336	CO-VALSACOR, film-coated tablets, 28 tablets, 5909990740277	
337	OLFEN 75, solution for injection, 5 ampules a 2ml, 5909990161119	
338	EUTHYROX N 100, tablets, 100 tablets , 5909991051426	
339	ROSWERA, film-coated tablets, 28 tablets, 5909990895533	
340	VESSEL DUE F, soft capsules, 50 capsules, 5909990039616	
341	CAPTOPRIL POLFARMEX, tablets, 30 tablets , 5909990830817	
342	CLEMASTINUM AFLOFARM, syrup, 100 ml, 5909990955510	
343	THEOSPIREX RETARD, film-coated prolonged-release tablets, 50 tablets, 5909990803910	
344	TIALORID, tablets, 50 tablets, 5909990206025	
345	ZAFIRON, inhalation powder, hard capsules, 120 capsules, 5909990937981	
346	AMOKSIKLAV, powder for oral suspension, 140 ml (35 g), 5909990894833	
347	NOLPAZA 20, gastro-resistant tablets, 56 tablets , 5909990075041	
348	ACIDOLAC BABY, oral drops, 10 ml, 5903060605626	

349	ATRODIL, pressurised inhalation, solution, 10 ml (200 actuations), 5909991185879	
350	AXTIL, tablets, 30 tablets , 5909990337958	
351	MEMOTROPIL, film-coated tablets, 60 tablets , 5909990873746	
352	HEVIRAN, film-coated tablets, 30 tablets , 5909990840113	
353	VIVACE 5 MG, tablets, 30 tablets , 5909990610495	
354	VIVACE 10 MG, tablets, 30 tablets , 5909990610532	
355	FUROCEF, film-coated tablets, 14 tablets, 5909991231217	
356	DEBRETIN, film-coated tablets, 100 tablets, 5909990927043	
357	RUPAFIN, oral solution, 120 ml , 5909990991921	
358	MELOXISTAD, tablets, 20 tablets, 5909990431168	
359	LISIPROL, tablets, 28 tablets , 5909990682447	
360	BIOSOTAL 40, tablets, 60 tablets , 5909990365715	
361	NONPRES, film-coated tablets, 30 tablets, 5909991014728	
362	SUMAMED FORTE, powder for oral suspension, 30 ml , 5909990742226	
363	NOLPAZA 40, gastro-resistant tablets, 28 tablets , 5909990075089	
364	AMLOPIN 10 MG, tablets, 30 tablets , 5909990048977	
365	HELICID 20, gastro-resistant capsules, hard, 28 capsules , 5909990420629	
366	ROTICOX, film-coated tablets, 30 tablets, 5909991313104	
367	ROMAZIC, film-coated tablets, 30 tablets, 5909990919659	
368	RUPAFIN 10, tablets, 15 tablets , 5909990083268	
369	XARELTO 20, film-coated tablets, 28 tablets, 5909990910724	
370	NITRENDYPINA EGIS, tablets, 60 tablets, 5909990694754	
371	AVAMYS, nasal spray, suspension, 120 actuations, 5909990076765	
372	ATORVASTEROL, film-coated tablets, 30 tablets, 5909990078028	
373	ACTELSAR, tablets, 28 tablets, 5909990891863	
374	KIDOFEN DUO, oral suspension, 100 ml, 5909991067496	
375	ACTELSAR, tablets, 28 tablets, 5909990891832	
